# KTaO_3_‑Based Supercurrent Diode

**DOI:** 10.1021/acs.nanolett.5c05590

**Published:** 2026-01-21

**Authors:** Muqing Yu, Jieun Kim, Ahmed Omran, Zhuan Li, Jiangfeng Yang, Sayanwita Biswas, Chang-Beom Eom, David Pekker, Patrick Irvin, Jeremy Levy

**Affiliations:** † Department of Physics and Astronomy, 6614 University of Pittsburgh, Pittsburgh, Pennsylvania 15260, United States; ‡ Department of Materials Science and Engineering, 5228 University of Wisconsin—Madison, Madison, Wisconsin 53706, United States

**Keywords:** supercurrent diode effect, KTaO_3_, oxide interface, c-AFM lithography, vortex dynamics

## Abstract

The supercurrent diode effect (SDE), characterized by nonreciprocal critical currents, represents a promising building block for future dissipationless electronics and quantum circuits. Realizing SDE requires breaking both time-reversal and inversion symmetry in the device. Here we use conductive atomic force microscope (c-AFM) lithography to pattern reconfigurable superconducting weak links (WLs) at the LaAlO_3_/KTaO_3_ (LAO/KTO) interface. By deliberately engineering the WL geometry at the nanoscale, we realize SDE in these devices in the presence of modest out-of-plane magnetic fields. The SDE polarity can be reversed by simply changing the WL position, and the rectification efficiency reaches up to 13% under optimal magnetic field conditions. Time-dependent Ginzburg–Landau simulations reveal that the observed SDE originates from asymmetric vortex motion in the inversion-symmetry-breaking device geometry. This demonstration of SDE in the LAO/KTO system establishes a versatile platform for investigating and engineering vortex dynamics, forming the basis for engineered quantum circuit elements.

## Graphical abstract

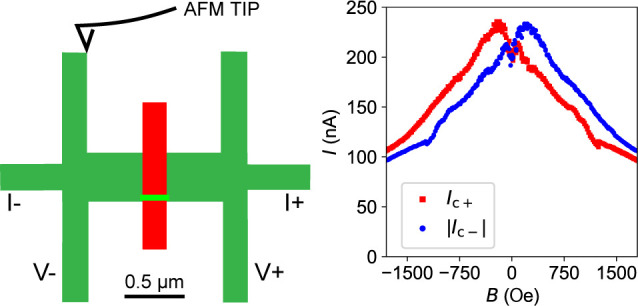

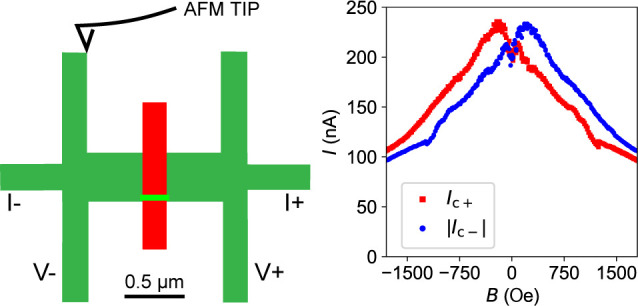

The supercurrent diode effect (SDE) refers to the nonreciprocal current flow in a superconductor, where its critical current differs significantly depending on the direction of the current. This asymmetry, analogous to semiconductor diodes, enables the rectification of alternating currents in superconducting circuits and represents a useful component for low-dissipation quantum electronics. The SDE has been reported in various material platforms,^1–10^ with multiple theoretical explanations proposed.^11–14^ Although the exact mechanism varies between systems, two necessary ingredients are common to all reports. First, inversion symmetry must be broken in either the crystal structure or the device geometry. Second, time-reversal symmetry must be broken by an external magnetic field^1,2^ or internally by spontaneous magnetic order.^6,7^ The diode rectification efficiency η is defined as $\eta =\frac{I_{c+}-\left|I_{c-}\right|}{I_{c+}+\left|I_{c-}\right|}$ , where *I*_c+_ and *I*_c–_ are the critical currents in opposite directions. The sign and magnitude of η indicate the polarity and strength of the SDE, respectively. Realizing SDE with controllable polarity and high rectification efficiency remains an active area of research. KTaO_3_ (KTO), specifically its heterointerface with LaAlO_3_ (LAO), has recently emerged as a platform for studying two-dimensional (2D) superconductivity.^15,16^ The 111-oriented LAO/KTO interface exhibits superconductivity with *T*_c_ up to 2 K (ref 15) while offering exceptional flexibility through conductive atomic force microscope (c-AFM) lithography. Nanoscale superconducting devices can be written and erased in a reconfigurable manner by sketching on the LAO/KTO surface with a biased AFM tip.^17–20^ Superconducting weak links (WLs), essential components for superconducting circuits, were previously realized on LAO/KTO.^19^ In this work, we report the SDE in c-AFM-patterned KTO WLs when time-reversal symmetry is broken by an external magnetic field and inversion symmetry is broken by deliberately displacing the WL from the centerline of the device. We demonstrate control over both the polarity and the magnitude of SDE by varying the WL position. The rectification efficiency |η| reaches up to approximately 13% under optimal magnetic field conditions. Previous studies have highlighted the critical role of vortex dynamics in the SDE of 2D superconductors.^14,21,22^ Through time-dependent Ginzburg–Landau (TDGL) simulations, we ascribe the origin of the SDE to the combination of Meissner screening currents and asymmetric vortex surface barriers in the KTO WLs. Two of the studied WLs exhibit SDE combined with enhanced superconductivity at finite magnetic fields, where both *I*_c+_ and |*I*_c–_| increase as the field deviates from zero. One possibility is that this effect is caused by the magnetization of local magnetic moments in the KTO sample.^23–25^ Alternatively, this could be a signature of quantization of the number of vortices in the device, that is, the Weber blockade.^26,27^

We report six WLs (Devices A–F) patterned by c-AFM lithography at the LAO/KTO (111) interface (see the Methods section in the Supporting Information). Devices B–F exhibit a clear SDE under finite magnetic fields applied perpendicular to the device plane (*B* = *B*_*z*_), while Device A serves as a reference with suppressed SDE magnitude.

Device A (Figure 1a) exemplifies an inversion-symmetric WL geometry. A horizontal 2D superconducting channel (width *w* = 400 nm) is divided into left and right halves and then bridged by a superconducting nanowire (WL) at the center. The WL is positioned at the vertical center of the channel, equidistant from both edges. Details of the c-AFM lithography process are provided in ref 19 and in the Methods section of the Supporting Information. During cryogenic measurements at *T* = 50 mK, a magnetic field perpendicular to the sample plane is applied. Current–voltage (*I*–*V*) measurements of Device A at *B* = −500 and +500 Oe are shown in the top and bottom panels of Figure 1d, respectively. At both field values, *V* remains at zero as *I* increases from zero until an abrupt transition to the normal state occurs at the positive critical current *I*_c+_ (or positive switching current). As *I* decreases from its positive maximum back to zero, the device returns to the superconducting state at the positive retrapping current *I*_r+_ (red curves, Figure 1d). Similarly, the negative critical current *I*_c–_ and negative retrapping current *I*_r–_ are observed as *I* sweeps from zero in the negative direction and back (blue curves, Figure 1d). The hysteretic *I*–*V* characteristics indicate that the WL operates in the underdamped regime or experiences self-heating in the normal state. At *B* = ±500 Oe, Device A exhibits minimal diode rectification because *I*_c+_ and |*I*_c–_| differ by <3 nA, which corresponds to |η| < 1%.

**Figure 1:**
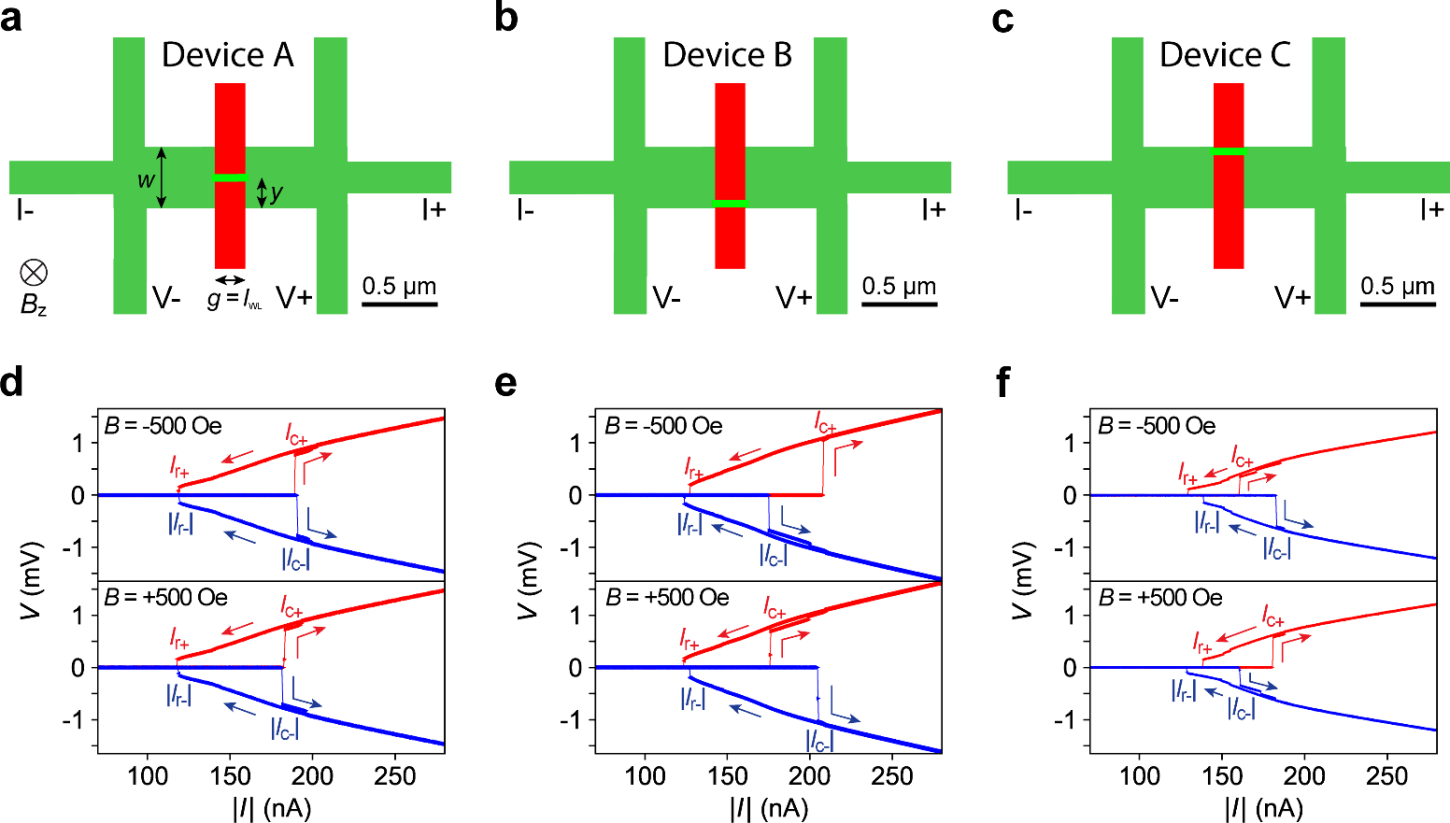
SDE in KTO WL Devices A–C. (a) Layout of the reference Device A. Device A is created by cutting a 2D channel (dark green, width *w* = 400 nm) into the left and right halves by the red rectangle and then bridging them with a nanowire (light-green path), which serves as the WL. The gap *g* created by the cutting corresponds with the length of the WL: *l*_WL_ = *g*, which is estimated to be ≈200 nm (see Supplementary Note S2). The WL is centered in the vertical direction. We define *y* as the vertical distance between the center of the WL to the bottom edge of the 2D channel, which equals *w*/2 = 200 nm. *I*+, *I*–, *V*+, and *V*– indicate the current source, current drain, and the two voltage leads used in the following four-terminal *I*–*V* measurements. Positive bias current (*I* > 0) flows from the right to the left. The positive magnetic field (*B* > 0) points toward the sample plane. (b) Layout of Device B, where the WL is placed close to the bottom edge of the 2D channel (*w* = 400 nm; *y* = 32 nm). (c) Layout of Device C, where the WL is placed close to the top edge of the 2D channel (*w* = 400 nm; *y* = 368 nm). (d) *I*–*V* measurements of Device A at *B* = −500 Oe (top) and *B* = +500 Oe (bottom). The red curve is the *V* vs *I* curve under positive current (*I* > 0), while the blue curve is the *V* vs |*I*| curve under negative current (*I* < 0). The arrows indicate the current sweep directions, while the switching currents *I*_c+_ and |*I*_c–_| and the retrapping currents *I*_r+_ and |*I*_r–_| are labeled. (e) *I*–*V* measurements of Device B, where an obvious mismatch between *I*_c+_ and |*I*_c–_| can be observed. At *B* = −500 Oe, *I*_c+_ > |*I*_c–_|, while at *B* = +500 Oe, *I*_c+_ < |*I*_c–_|. (f) *I*–*V* measurements of Device C. At *B* = −500 Oe, *I*_c+_ < |*I*_c–_|, while at *B* = +500 Oe, *I*_c+_ > |*I*_c–_|. Note that all plots in this figure were taken at *T* = 50 mK with a backgate voltage *V*_bg_ = −30 V applied on Devices A–C.

The values of *I*_c+_ and |*I*_c–_| deviate from each other when the inversion symmetry is deliberately broken in the device layout by displacing the WL to one side of the channel. Device B is patterned identically to Device A except that the WL is positioned near the bottom edge of the channel (distance from the bottom: *y* = 32 nm; Figure 1b). Under a negative magnetic field *B* = −500 Oe, Device B exhibits a pronounced SDE, with *I*_c+_ exceeding |*I*_c–_| by approximately 30 nA (η = +8.3%; Figure 1e, top panel). When *B* switches to +500 Oe, the SDE polarity in Device B changes sign, as *I*_c+_ now falls below |*I*_c–_| by approximately 30 nA (η = −8.0%; Figure 1e, bottom panel). Remarkably, the SDE polarity can be flipped by “flipping” the WL position. In Device C, the position of the WL is shifted from the bottom to the top of the 2D channel (*y* = 368 nm; Figure 1c) relative to Device B. This results in *I*_c+_ < |*I*_c–_| at *B* = −500 Oe (η = −6.4%; Figure 1f, top panel), while *I*_c+_ > |*I*_c–_| at *B* = +500 Oe (η = +6.5%; Figure 1f, bottom panel), opposite to the behavior of Device B.

Continuous magnetic field sweeps provide additional information about how *I*_c±_ and the SDE strength evolve with *B*. Parts a–c of Figure 2 present intensity plots of d*V*/d*I* as a function of *I* and *B* for Devices A–C, respectively. These plots take the portion of *I*–*V* curves where the magnitude of current |*I*| increases from 0. This enables us to visualize and extract *I*_c±_ at the points where d*V*/d*I* increases above *R*_N_/2 (half of the normal state resistance). We note that in all of the following d*V*/d*I* intensity plots, |*I*| increases from 0 if not specifically labeled. The d*V*/d*I* pattern of Device A (reference device) appears to be symmetric with respect to *I* = 0 (Figure 2a), with *I*_c+_ and |*I*_c–_| nearly overlapping across the entire measured field range (Figure 2d). In contrast, the d*V*/d*I* pattern of Device B appears skewed (Figure 2b), with a clear deviation between the two critical currents: *I*_c+_ < |*I*_c–_| at *B* > 0 and *I*_c+_ > |*I*_c–_| at *B* < 0 (Figure 2e). Despite this skewness, d*V*/d*I* and *I*_c_ of Device B follow inversion symmetry with respect to field *B* and bias *I*:

1 $$dV/dI{|}_{I,B}=dV/dI{|}_{-I,-B}$$

2 $$I_{c+}\left(B\right)=-I_{c-}\left(-B\right)$$

These relationships are theoretically expected and experimentally observed in systems without intrinsic time-reversal symmetry breaking.^10^ As shown in Figure 2h, η of Device B transitions from positive to negative approximately linearly with *B* as the field increases from −200 to +200 Oe. The efficiency reaches its maximum η_max_ = +12.0% at the optimal field *B*_η_max__ = −1198 Oe and its minimum η_min_ = −12.4% at *B*_η_min__ = +1242 Oe (Figure 2h). At |*B*| > 1500 Oe, the difference between *I*_c+_ and |*I*_c–_| is suppressed, reducing |η| accordingly. We note that η_max_ ≈ −η_min_ and *B*_η_max__ ≈ −*B*_η_min__ are due to the symmetry expressed in eq 2. For Device C, with the WL positioned on the opposite edge of the channel, the d*V*/d*I* vs *I* vs *B* pattern (Figure 2c) and critical currents *I*_c±_(*B*) (Figure 2f) exhibit opposite skew compared to Device B. Device C achieves η_max_ = +9.75% at *B*_η_max__ = +853 Oe and η_min_ = −9.04% at *B*_η_min__ = −853 Oe (Figure 2i). In Devices B and C, there is also a slight mismatch between the positive and negative retrapping currents (Figure S1), but it is less significant than the difference between *I*_c+_(*B*) and *I*_c–_(*B*). For the reference Device A, with its inversion-symmetric layout, η­(*B*) remains confined within ±3% across the entire field range (Figure 2g).

**Figure 2:**
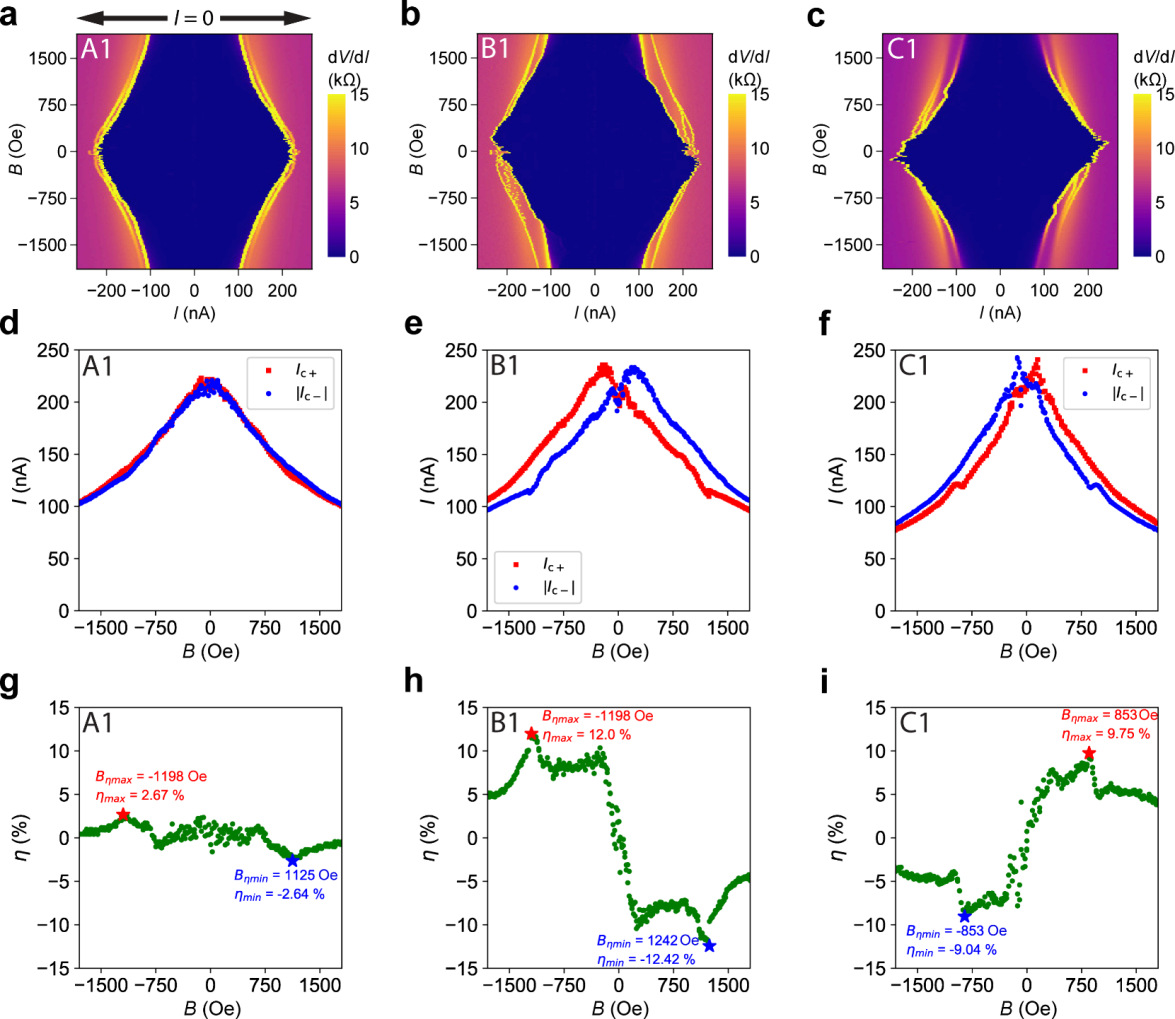
Magnetic field sweep of Devices A–C. Panels a–c show the intensity plots of differential resistance d*V*/d*I* vs *I* vs *B* for Devices A–C, respectively. We note that, in these plots, current *I* sweeps from *I* = 0 to |*I*| > 0 to capture the switching behavior from the superconducting state to the normal state. Panels d–f display the extracted switching currents *I*_c±_ as a function of *B* for Devices A–C, respectively. Panels g–i show the extracted diode efficiency η as a function of *B* for Devices A–C. On the top left corner of each panel, the label consists of a letter that indicates the corresponding device and a number that points to the measurement configuration (mapping in Figure S7). All measurements were performed at *T* = 50 mK with a backgate voltage *V*_bg_ = −30 V.

A comparison of Devices A–C reveals two key findings. First, a strong SDE in KTO WLs requires breaking of both the time-reversal symmetry through the applied field *B* and the inversion symmetry through the device geometry. Second, the sign of η can be controlled by varying the WL position. Electrostatic gating is applied on Devices A–C by a voltage *V*_bg_ on the backside of the sample.^16,28^ The effect of *V*_bg_ on the SDE is discussed in Supplementary Note S1. Device F, created by using an alternative c-AFM process in which the channel is only partially cut to leave a thin conducting path near the bottom edge (Figure S5), also demonstrates SDE with the same polarity as Device B, providing an alternative approach to KTO supercurrent diode patterning.

Two additional supercurrent diodes, Devices D and E, were created by c-AFM lithography. As shown in Figure 3a, inversion symmetry is again broken in these devices by positioning the WL near the bottom edge in Device D (*y* = 36 nm) and near the top edge in Device E (*y* = 364 nm). Their *I*–*V* characteristics at *T* = 50 mK and *B* = ±500 Oe again show asymmetry between the positive and negative critical currents (Figure 3b,c), with hysteretic behavior and distinct retrapping currents (Figure S12). The SDE polarity in Device D matches that of Device B, with both exhibiting *I*_c+_ > |*I*_c–_| at *B* < 0 and *I*_c+_ < |*I*_c–_| at *B* > 0 (Figure 3e). Conversely, Devices C and E show consistent behavior with *I*_c+_ < |*I*_c–_| at *B* < 0 and *I*_c+_ > |*I*_c–_| at *B* > 0 (Figure 3h). This further confirms that the WL position is the decisive factor determining the SDE polarity. The efficiency η of Devices D and E reaches η_max_ > +10% and η_min_ < −10% (Figure 3f,i), comparable to the η_max(min)_ measured in Devices B and C.

**Figure 3:**
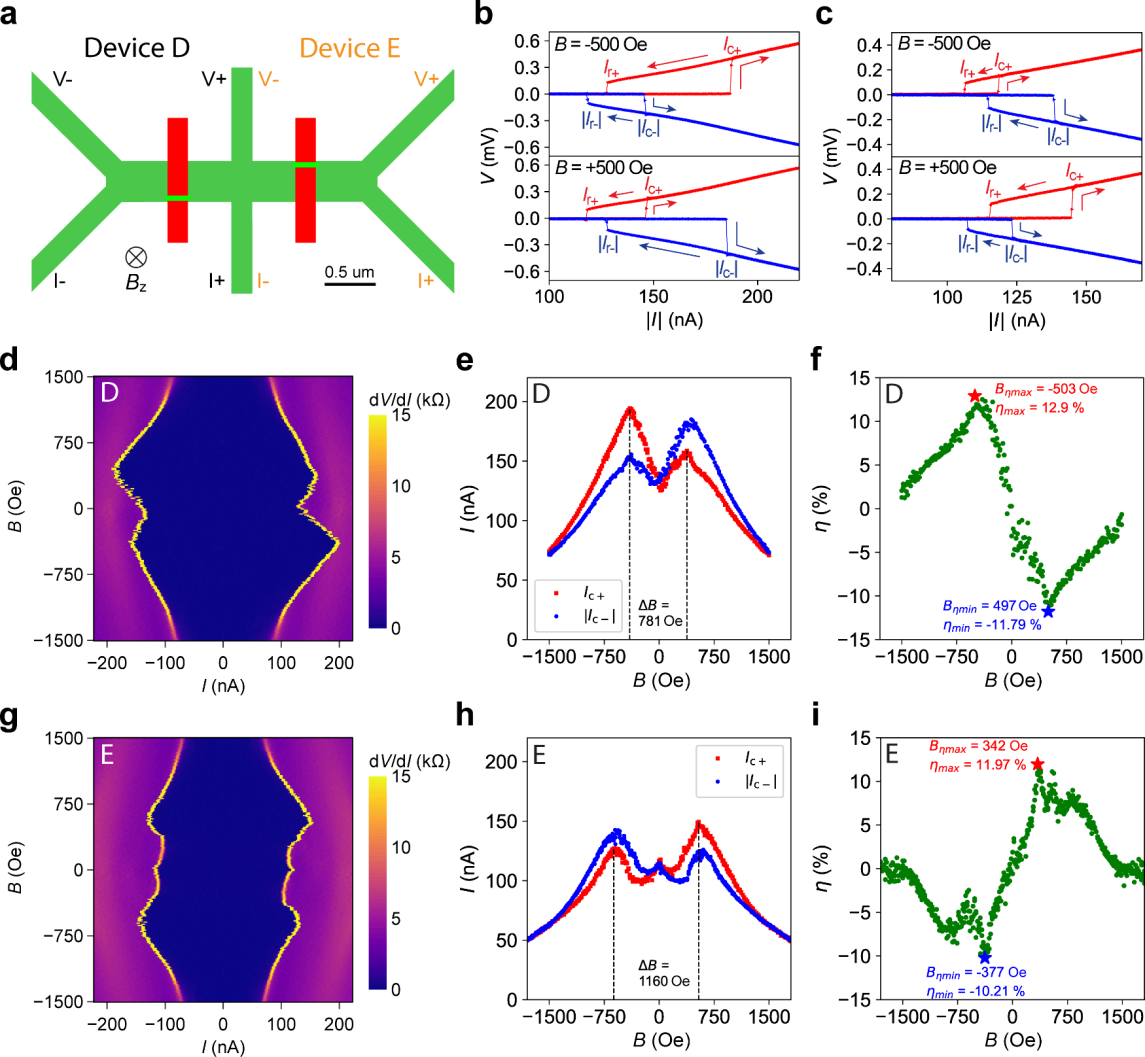
Supercurrent diode Devices D and E with slanted M-shaped *I*_c_ vs *B* patterns. (a) Device layout. Devices D and E were patterned together in one run by c-AFM lithography. Both WLs are put in the *w* = 400 nm 2D channel with WL Device D (E) positioned at *y* = 36 nm (*y* = 364 nm). The measurement configuration for Device D (E) is indicated in black (orange) *I*+/*I*–/*V*+/*V*– labels, as two leads are shared between them. (b and c) *I*–*V* measurements of Device D (E) at *B* = ±500 Oe. (d) d*V*/d*I* vs *I* vs *B* intensity plot of Device D. (e) *I*_c±_ vs *B* of Device D, which follows a slanted “M” pattern. Black dashed lines label the two *I*_c+_ maxima at *B* = +400 and −380 Oe, while *I*_c+_(*B* = 0) is lower than either of these maxima. (f) η vs *B* relationship of Device D. (g–i) d*V*/d*I* vs *I* vs *B* intensity plot and *I*_c±_ vs *B* and η vs *B* relationships of Device E. In panel h, the two *I*_c+_ maxima occur at *B* = +540 and −620 Oe. All plots in this figure were taken at *T* = 50 mK with backgate grounded *V*_bg_ = 0 V on Devices D and E.

Beyond this asymmetry, the field-dependent evolution of *I*_c+_(*B*) and *I*_c–_(*B*) in these devices warrants attention. In Device D, rather than the typical suppression of *I*_c_ by a magnetic field, *I*_c+_ increases from 135 nA at *B* = 0 to 160 nA at *B* = +380 Oe (red data points, Figure 3e). This constitutes a local *I*_c+_ peak on the *B* > 0 side, after which *I*_c+_ decreases with a further field increase. When *B* decreases from zero in the negative direction, *I*_c+_ again increases from 135 nA, reaching *I*_c+_ = 190 nA at *B* = −400 Oe before decreasing. We define the field separation between the two *I*_c+_ peaks as Δ*B* = 780 Oe. A similar enhancement of *I*_c_ at a finite field occurs in Device E, whose *I*_c+_(*B*) exhibits two peaks away from *B* = 0. The slanted M-shaped *I*_c_(*B*) patterns in Devices D and E result from the combination of two effects. First, the slant originates from the SDE. Second, the M shape reflects enhanced *I*_c_ by magnetic field, and we discuss its possible origin in later paragraphs.

The observed SDE in KTO WLs exhibits two characteristic behaviors: the effect reverses the sign upon reversing the out-of-plane magnetic field and upon repositioning the WL from one edge to the other. These observations suggest that Meissner currents play a central role in the SDE mechanism. Previous studies of SDE in thin metallic superconducting films have highlighted the importance of Meissner currents and provide a framework for our analysis.^14,21^ In 2D superconducting systems, dissipation typically arises from vortex motion rather than Cooper pair breaking. To visualize vortex dynamics in our KTO WL geometry, we perform TDGL simulations (see ref 29 and the Methods section in the Supporting Information). The simulated device consists of a vertical channel (*w* = 400 nm) with a narrow constriction near its left edge, constituting the WL (Figure 4a). The current source (drain) is defined at the top (bottom) edge of the channel. The simulated WL dimensions are *l*_WL_ = 200 nm and *w*_WL_ = 50 nm, which are estimated based on Device F characteristics, as detailed in Supplementary Note S2. By design, it closely resembles Device C (Figure 1c) rotated counterclockwise by 90°.

**Figure 4:**
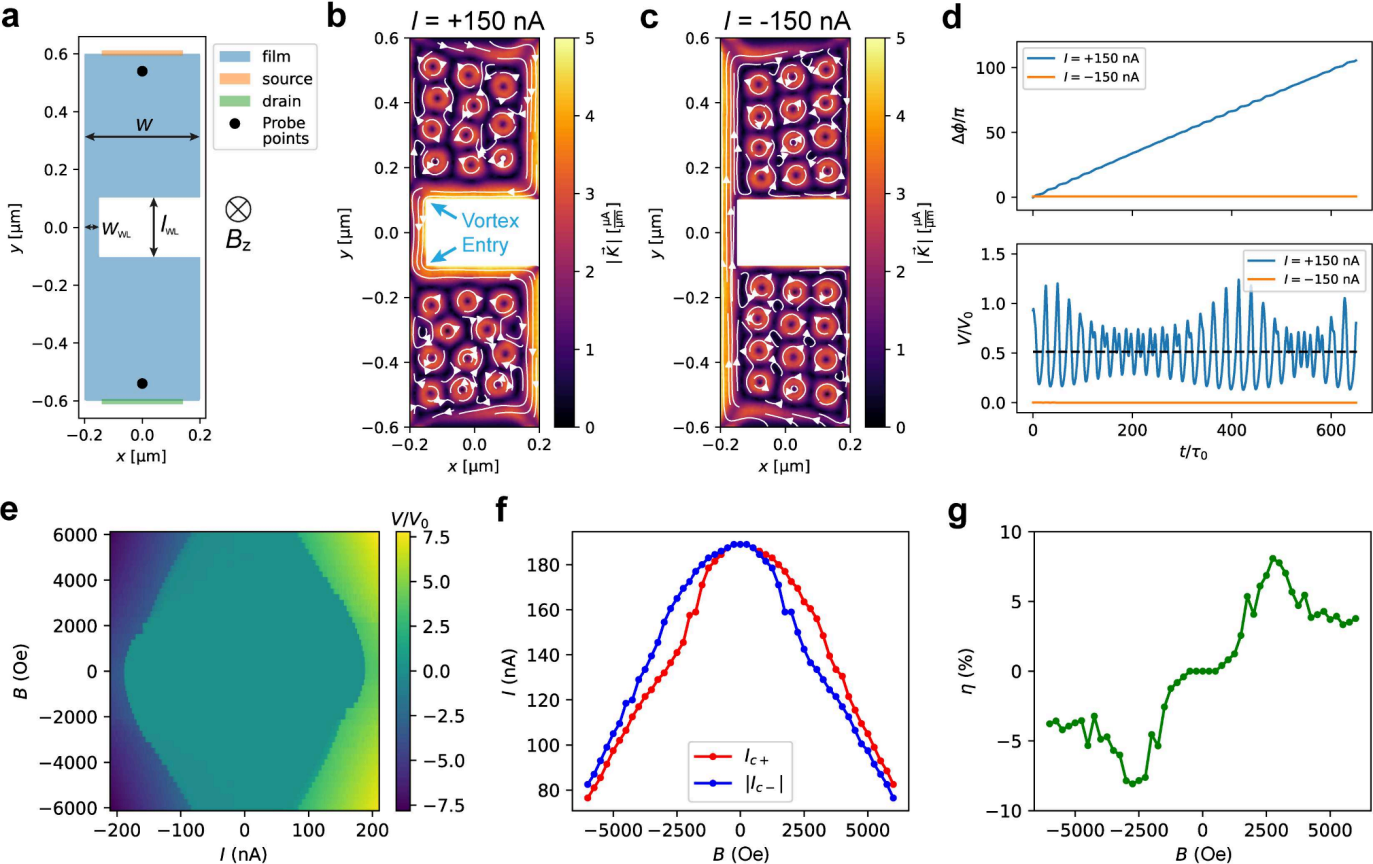
Simulations of KTO WLs using TDGL theory. (a) Device geometry used in the TDGL simulation. The 2D channel (blue) has width *w* = 400 nm and length *l* = 1200 nm. On its top and bottom edges, there are the current source and drain, indicated by the orange and green bars, respectively. Positive current *I* > 0 flows from the top to the bottom. The narrow constriction (WL) has width *w*_WL_ = 50 nm and length *l*_WL_ = 200 nm, located near the left edge of the 2D channel. The phase difference and voltage between the two black dots are output by TDGL calculations. We define a positive magnetic field (*B* > 0) as pointing into the sample plane, same as the experimental setup. (b) Current density *K*(*x*,*y*) calculated under the conditions *B* = −2000 Oe and *I* = +150 nA. The color scale indicates the magnitude of *K*, while white arrows indicate its direction. The two blue arrows point to the two locations with relatively low surface barriers for vortex entry. (c) *K*(*x*,*y*) calculated at *B* = −2000 Oe and *I* = −150 nA. (d) Evolution of phase difference Δϕ and voltage *V* as a function of time. Black dashed line: time-averaged voltage under *I* = +150 nA bias. (e) *V* vs *I* vs *B* intensity plot from pyTDGL simulation. (f) *I*_c±_ vs *B* relationship extracted from the simulated *I*–*V* curves. (g) η vs *B* relationship extracted from part f.

Parts b and c of Figure 4 show the calculated current density *K*(*x*,*y*) under *B* = −2000 Oe with applied currents of *I* = +150 and −150 nA, respectively. Upon the introduction of *B* = −2000 Oe, the Meissner effect induces a clockwise screening current. Also, some 11–12 vortices are introduced in the top and bottom halves of the device, represented by where *K* forms circles. These vortices are static and therefore do not contribute to dissipation (see Supplementary Video 1 and Supplementary Video 2). The total current density is the sum of the applied current and the clockwise screening current, resulting in enhanced (suppressed) |*K*| along the right (left) edge of the device under *I* = +150 nA bias (Figure 4b). With the supercurrent concentrating at the right edge of the device, the two corners near the WL (marked by arrows in Figure 4b) act as gateways for vortex entry due to the low surface barrier in these highly curved regions. After entry, these mobile vortices traverse the WL and exit at the left edge (Supplementary Video 1). Each vortex traveling from right to left causes the phase difference Δϕ between the top and bottom of the device to evolve by 2π, corresponding to a voltage peak (blue curves, Figure 4d). The time-averaged voltage is indicated by the black dashed line in Figure 4d, which would be the measurable direct-current (dc) voltage in the experiments.

In contrast, a negative bias combined with the clockwise screening current produces enhanced (weakened) current density on the left (right) side of the device (Figure 4c). In this scenario, the supercurrent concentrates at the left edge, which is relatively flat and thus presents a higher energy barrier for vortex entry. At *I* = −150 nA throughout the simulated time frame, no mobile vortex enters the device (Supplementary Video 2), preventing phase slips and maintaining *V* = 0 (orange curves, Figure 4d). The asymmetric vortex surface barriers at the two edges, combined with Meissner currents, lead to dissipation onset under *I* = +150 nA but not under *I* = −150 nA. By performing TDGL calculations across a range of bias currents *I* and magnetic fields *B* and extracting the time-averaged dc voltage at each point (see the Methods section in the Supporting Information), we obtain the skewed *V* vs *I* vs *B* pattern in Figure 4e. We note that simulated *I*_c±_ exhibit *I*_c+_(*B*) < |*I*_c–_(*B*)| for *B* < 0 and *I*_c+_(*B*) > |*I*_c–_(*B*)| for *B* > 0 (Figure 4f), consistent with the SDE polarity of Device C. At *B* = 0, the SDE has the sign reversal due to the direction change of the Meissner current under *B* > 0 vs *B* < 0. The simulated *I*_c±_(*B*) also follows the inversion symmetry expressed in eq 2. Regarding efficiency, the calculated η reaches its maximum and minimum of η_max_ = −η_min_ = +8.1% at fields *B*_η_max__ = −*B*_η_min__ = +2750 Oe (Figure 4g). The experimentally measured η_max_ and |η_min_| of Devices B–F range between 9% and 13%, very close to the calculated value. Meanwhile, the measured *B*_η_max__ and |*B*_η_min__| are scattered, ranging from 300 to 1300 Oe, lower than the calculated results, which can be attributed to errors in the material parameters used in the simulation (see the Methods section in the Supporting Information). Inhomogeneities or defects within the KTO sample and fluctuations during c-AFM lithography can introduce random asymmetries and disorder, such as rough edges and vortex pinning centers, which can also cause the discrepancy between the measured and simulated *B*_ηmax(min)_. The same randomly occurring asymmetries lead to the weakened but nonzero SDE in the reference Device A (|η| < 3%; Figure 2d). The TDGL simulation not only highlights two essential ingredients for the SDE (Meissner currents and asymmetric vortex surface barriers at the two edges^14,21^ ) but also demonstrates approximate quantitative agreement with the observed SDE strength in our KTO WLs.

During the measurements of Devices A–C, we observed that the *I*_c±_(*B*) patterns depend on the choice of the measurement configuration. We attribute this to the alternation of the current profile and vortex surface barriers upon changing the position of current leads, which is verified qualitatively by TDGL simulation in Supplementary Note S3.

In Devices D and E, *I*_c_(*B*) exhibits two peaks (separated by Δ*B*), where *I*_c_ exceeds its zero-field value, roughly following a slanted M shape. Previous studies reported the enhancement of superconductivity by magnetic fields in Zn, MoGe, and Nb superconducting nanowires.^25,30^ Specifically, ref 25 demonstrated an M-shaped *I*_c_(*B*) relationship bearing great similarity to our observations, which was attributed to spin-exchange scattering between Cooper pairs and magnetic moments on the nanowire surfaces. We believe that a similar mechanism can qualitatively explain our observations in KTO WLs. At *B* = 0, local magnetic moments in the KTO sample induce exchange scattering of electrons, effectively breaking Cooper pairs and weakening superconductivity.^23–25^ Consequently, vortex entry and nucleation become more energetically favorable, suppressing *I*_c_. At finite *B*, the magnetic moments are aligned by the field and exchange scattering is quenched, leading to higher *I*_c_. At high *B*, conventional orbital and Zeeman effects dominate, weakening the superconductivity and causing *I*_c_ to decrease. Competition between magnetic moment pair breaking and orbital and Zeeman pair breaking can produce a nonmonotonic, M-shaped *I*_c_(*B*) curve. Superimposed with SDE, this yields the slanted M-shaped *I*_c_(*B*) curves observed.

The fact that magnetic moments are distributed unevenly in the KTO sample explains the absence of M shape in *I*_c_(*B*) of Devices A–C. Several reports^31,32^ have suggested magnetism in the LAO/KTO system, which is ascribed to Ta 5d states and O vacancies. Magnetic impurities such as Fe and Ni can also be brought into the sample, especially during the polishing process of the crystal.^33^ Electrostatic gating with a backgate (*V*_bg_) is performed on Devices D and E (Figures S13 and S14). The magnitude of SDE η_max(min)_ is barely affected by *V*_bg_, always maxing out at ≈10% (top panel, Figure S15), similar to the case of Devices A–C (discussed in Supplementary Note S1). Meanwhile, Δ*B* that separates the two *I*_c_ peaks increases monotonically as *V*_bg_ decreases (bottom panel, Figure S15), which suggests that exchange scattering may be more pronounced with lower superfluid density and higher disorder. Enhancement of *I*_c_ by the field persists at temperature *T* = 500 mK (Device D, Figure S16b). At *T* = 900 mK, however, *I*_c_(*B*) loses its M shape and only SDE can be seen (Figure S16e). Further increases in *T* greatly suppress the superconductivity and smear out the SDE (Figure S16h). This temperature dependence indicates that the onset of exchange scattering may occur at a temperature ∈ (500 mK, 900 mK), lower than the superconducting critical temperature *T*_c_.

Another possible origin for the nonmonotonic *I*_c_(*B*) relationship is the Weber blockade of vortices.^26,27^ Under vortex–charge duality, the WL can be viewed as a vortex analogue of a Coulomb-blockaded quantum dot. As field *B* changes, the device periodically enters and exits the “blockaded” states with a fixed number of vortices, resulting in *I*_c_ oscillations as a function of *B*.^26^ *I*_c_(*B*) oscillations can also be seen in Devices A–C under certain measurement configurations (Figures 2f, S9, and S10).

The demonstration of a geometrically engineered, reconfigurable SDE at the LAO/KTO interface establishes a uniquely versatile platform for both fundamental physics and quantum technologies. The ability to write, erase, and rewrite WLs with c-AFM lithography enables the rapid prototyping of nonreciprocal circuit elements for dissipationless electronics. This precise geometric control also enables on-demand engineering of the energy landscape of vortices, making the system an ideal laboratory for systematic studies of 2D vortex dynamics. For example, a vortex pinning center may be defined by simply engaging a negatively biased AFM tip on the device. Furthermore, the large permittivity of the KTO substrate enables electrostatic gating as a convenient tuning nob for devices. This, along with the large kinetic inductance of the LAO/KTO interface,^34,35^ results in slow light speed in the system, which is crucial for compact circuit elements. This work positions the LAO/KTO system at the forefront of research in 2D SDE, vortex physics, and the next generation of quantum circuits.

## Supplementary Material







## Notes

Methods section, as well as Supplementary Notes 1–3 and Supplementary Figures S1–S16, which provide additional experimental details (PDF)
Supplementary Video 1 showing the animated vortex motion under *I* = +150 nA bias from the TDGL simulation (MP4)
Supplementary Video 2 showing the animated vortex motion under *I* = −150 nA bias from the TDGL simulation (MP4)
