# Supplementary material for: KTaO3‑Based Supercurrent Diode
Source: Nano Lett. 2026 Jan 21;26(8):2869–76. doi: 10.1021/acs.nanolett.5c05590 (PMC12964540; doi:10.1021/acs.nanolett.5c05590)
Supplement: Supplementary file 1 [file nl5c05590_si_001.pdf]

Supporting Information for:

$KTaO_3$-Based Supercurrent Diode

Muqing Yu[1], Jieun Kim[2], Ahmed Omran[1], Zhuan Li[1], Jiangfeng Yang[2], Sayanwita Biswas[1], Chang-Beom Eom[2], David Pekker[1], Patrick Irvin[1], Jeremy Levy[1,*]

[1]Department of Physics and Astronomy, University of Pittsburgh, Pittsburgh, PA 15260, USA

[2]Department of Materials Science and Engineering, University of Wisconsin-Madison, Madison, WI 53706, USA

*Corresponding author. Email: jlevy@pitt.edu

This PDF file includes:

Methods

Supplementary Notes 1-3

Figures S1 to S16

Methods

Growth of LAO on KTO (111) substrate

The $LaAlO_3$ growth on $KTaO_3$ (111) substrate is carried out by pulsed laser deposition (PLD) with substrate heater temperature at 673 K in a dynamic oxygen pressure of 10^{-5} torr. The laser has fluence of 1.6 J/cm^2 and repetition rate of 1 Hz (248 nm, LPX 300, Coherent). $LaAlO_3$ is deposited from a single-crystal $LaAlO_3$ target (Crystec) with a target-to-substrate distance of 65 mm. The growth rate of $LaAlO_3$ is approximately 0.11 Å per laser pulse. Following the growth of 4.4 nm of LAO, the samples are cooled to room temperature by quenching in the growth atmosphere.

Conductive Atomic Force Microscope lithography

We closely follow the c-AFM lithographic process in Ref. 19 to create WLs, except for the specific tip voltage V_{tip}. Here, the 2D channel and the leads are written by $V_{tip} \in [+20\ \text{V}, +30\ \text{V}]$. Then the 2D channel is cut with $V_{tip} \in [-9\ \text{V}, -8\ \text{V}]$ for 3-4 times until no conductance is left between the two halves. Finally the WL is written once with $V_{tip} \in [+7\ \text{V}, +8\ \text{V}]$.

Current-voltage characteristics

Low-temperature $I-V$ characteristics are measured in a Quantum Design Physical Property Measurement System (PPMS) with a dilution refrigerator (DR) unit. In PPMS, B field perpendicular to the sample plane can be applied. Source voltages are output by National Instruments PXI-4461, which can perform both digital-to-analog and analog-to-digital conversion. Current biasing is achieved by shunting the device with 300 kΩ in-series resistance. The drain current and the voltages are measured after amplification by a Krohn-Hite 7008 multichannel preamplifier. When taking a single $I-V$ curve, the bias current I ramps from 0 to the positive maximum, then to the negative minimum, and finally back to 0. This way both the switching current I_c and the retrapping current I_r are captured. No averaging is performed between the $I-V$ curves, and each datapoint in the $I_c(B)$, $I_r(B)$ and $\eta(B)$ plots is extracted from a single $I-V$ curve.

Time-dependent Ginzburg Landau simulations

TDGL simulation is performed with the pyTDGL package (https://py-tdgl.readthedocs.io, Ref. 29), which we modified to incorporate thermal fluctuations. The modified codes are available in the github repository (https://github.com/pitt-pekker-group/TDGL-thermal-fluctuations). In the pyTDGL package we first choose the following parameters for LAO/KTO (111) interface: Ginzburg-Landau correlation length $\xi_{GL} = 20\,\text{nm}$, normal state conductiv-

ity $\sigma = 0.3\,\mathrm{S}/\mu\mathrm{m}$, London penetration depth $\lambda_L = 2.5\,\mu\mathrm{m}$, thickness of the LAO/KTO 2d electron gas (2DEG) $d = 5$ nm and reduced temperature $t = T/T_c = 0.05$. We justify these choices in the following:

(1) ξ_{GL} is extracted from the out-of-plane critical field $B_{c2}(T = 0) = \Phi_0/(2\pi\xi_{GL}^2)$ of a Hall-bar device in Figure S6. At $V_{bg} = 0$ V, $\xi_{GL} = 21.2\,\mathrm{nm}$ while at $V_{bg} = -60$ V, ξ_{GL} decreases to 16.2 nm. In the TDGL simulation we define $\xi_{GL} = 20\,\mathrm{nm}$ which is close to the measured value at $V_{bg} = 0$ V.

(2) The thickness d of the LAO/KTO(111) 2DEG has been calibrated in Ref. 15 to be 5.1 nm, and also reported by Ref. 16 to vary from 2 nm to 6 nm depending on the V_{bg} applied. Here we choose $d = 5\,\mathrm{nm}$ in our simulation.

(3) Normal state conductivity can also be extracted from Figure S6. The Hall-bar with 4:1 ratio has normal state resistance $R_N = 2.6$ kΩ at $V_{bg} = 0$ V and $R_N = 5$ kΩ at $V_{bg} = -60$ V. We choose the R_N at $V_{bg} = 0$ V, which gives sheet resistance $R_{sheet} = 650\ \Omega$, resistivity $\rho = R_{sheet}d = 3.25\ \Omega \cdot \mu\mathrm{m}$ and conductivity $\sigma = 1/\rho \approx 0.3$ S/μm.

(4) λ_L is related to the 3d superfluid density $n_{s,3d}$ in the following way: $\lambda_L^2 = m/(\mu_0 n_{s,3d} e^2)$. The 2d superfluid density of LAO/KTO(111) has been reported in Ref. 34 to be $n_{s,2d} = 2 \times 10^{12}$ cm^{-2}. Thus we can estimate $\lambda_L = \sqrt{md/(\mu_0 n_{s,2d} e^2)} \approx 2.5\ \mu$m.

(5) Thermal noise terms that depend on $t = T/T_c$ are included in the TDGL equations during simulation. T_c of the LAO/KTO(111) interface is known to be 1 to 2 K (Ref. 15 and Figure S16), and the $I-V$ measurements are performed in a PPMS setup with base temperature $T = 50$ mK, so t is chosen to be 0.05. We note that the actual electron temperature in our devices may be slightly higher than the cryostat temperature, which needs future noise thermometry to be accurately measured.

The pyTDGL package then generates the finite volume mesh for the device in main text Figure 4(a). We specify the maximum edge length to be 14 nm which is 70% of ξ_{GL}, resulting in ≈ 6000 mesh points. PyTDGL simulates how the order parameter ψ evolves at each

mesh point as a function of time. It outputs the phase difference $\Delta\phi(t)$ and voltage $V(t)$ across the two probe points we define. The time unit τ_0 of the horizontal axis in main text Figure 4(d) has the value $\tau_0 = \mu_0\sigma\lambda_L^2 = 2.4$ ps. and the voltage unit V_0 of the vertical axis has the value $V_0 = 2\Phi_0/(\pi\tau_0) = 0.56$ mV. At $B = -2000\,\mathrm{Oe}$ and $I = \pm150\,\mathrm{nA}$, the pyTDGL solver first goes through a "thermalization" step which lasts for $T_{therm} = 550\tau_0$, where the device is stabilized at the set field and current bias. Then the solver solves for a duration $T_{solve} = 650\tau_0$ while recording $\Delta\phi(t)$ and $V(t)$ to be plotted in main text Figure 4(d). The current density plots (main text Figure 4(b)(c)) are recorded at the timestamp $t = 100\tau_0$ during the solving step.

We run the pyTDGL solver at a series of current values to get a simulated $I - V$ curve: I from 0 nA to -210 nA with step of -1.5 nA, and then from 0 nA to +210 nA with step of 1.5 nA. With this I sequence we capture the switching current at both positive and negative bias to simulate the SDE strength correctly. At each current value, the solver thermalizes for $T_{therm} = 80\tau_0$ and then solve for $T_{solve} = 90\tau_0$. The mean voltage within this $90\tau_0$ solving time is recorded as the DC voltage to be plotted in main text Figure 4(e). The solution of the previous I is used as seed solution for the next I for faster thermalization. This $I - V$ curve simulation is then repeated at a series of B from $-6000\,\mathrm{Oe}$ to $6000\,\mathrm{Oe}$ with a step of $250\,\mathrm{Oe}$ to get the V vs I vs B plot (main text Figure 4(e)). We note the simulated $I_c(B = 0) = 190\,\mathrm{nA}$ (main text Figure 4(f)) agrees well with the experimentally measured $I_c(B = 0)$ from Devices A through F, which ranges from 120 nA to 210 nA.

Supplementary Note S1: Effect of electrostatic gating on the SDE of Devices A-C

KTO is known to be quantum paraelectric at cryogenic temperature,[28] which enables tuning of superfluid density and disorder in its two-dimensional electron gas by applying a voltage

V_{bg} on the backside of the sample.[16] We note the plots in Figure 2 of the main text are taken with $V_{\text{bg}} = -30$ V applied on Devices A-C. For each device, $I_{c\pm}(B)$ and $\eta(B)$ are also measured with $V_{\text{bg}} = -55$ V and with $V_{\text{bg}} = 0$ V, shown in Figure S2 and Figure S3 respectively. From the intensity plots of dV/dI versus I versus B (Figure S3(a)-(c), main text Figure 2(a)-(c), Figure S2(a)-(c)), we clearly observe the increase in dV/dI in the normal state when V_{bg} decreases from 0 V to -30 V to -55 V.

In terms of the diode behavior of each device, applying different V_{bg} does not change its SDE polarity. Device B always exhibits $\eta < 0$ at $B > 0$ and $\eta > 0$ at $B < 0$ (Figure S3(h), main text Figure 2(h), Figure S2(h)). Device C always shows the opposite polarity compared to Device B, with $\eta > 0$ at $B > 0$ and $\eta < 0$ at $B < 0$ (Figure S3(i), main text Figure 2(i), Figure S2(i)). The reference Device A always exhibits weaker SDE with $|\eta| < 4\%$ (Figure S3(g), main text Figure 2(g), Figure S2(g)).

However, V_{bg} can affect the specific $I_{c\pm}(B)$ and $\eta(B)$ patterns as well as extreme values of η. Figure S4 shows how η_{max} and η_{min} as well as the corresponding optimal B field $B_{\eta\text{max}}$ and $B_{\eta\text{min}}$ evolve with V_{bg}. Highest η_{max} and $|\eta_{\text{min}}|$ of Device C are achieved at $V_{\text{bg}} = 0$ V, while η_{max} and $|\eta_{\text{min}}|$ of Device B increase at negative V_{bg}. The parameters $B_{\eta\text{max}}$ and $B_{\eta\text{min}}$ of Device B change non-monotonically with V_{bg}. This inconsistency between Devices B and C prevents us from reaching any solid conclusion on how V_{bg} affects the diode performance.

Supplementary Note S2: Estimation of dimensions of KTO WLs

Devices A-E are patterned by cutting the two-dimensional conducting channel in two halves and then bridging them back together by writing a nanowire. In this section, we provide an estimation of length l_{WL} and width w_{WL} for the resulting WLs. Ref. 19 extracted the current-phase relationship (CPR) of KTO WLs by measuring quantum interference between two parallel WLs. From the CPR, l_{WL} is determined to be 200 to 300 nm, varying from

Device to device. The c-AFM lithographic process in this work closely follows Ref. 19, so we believe $l_{\mathrm{WL}} \in [200\,\mathrm{nm}, 300\,\mathrm{nm}]$ also applies to the WLs we create here. For the TDGL simulation described in main text Figure 4(a), we choose the lower bound $l_{\mathrm{WL}} = 200\,\mathrm{nm}$.

In terms of w_{WL}, we choose it to be 50 nm in the TDGL simulation, which can be justified by Device F shown in Figure S5. In Device F, instead of cutting the 2D channel completely in half, we leave a 60 nm gap at the bottom (Figure S5(a)), which is effectively a WL with $w_{\mathrm{WL,F}} \approx 60\,\mathrm{nm}$. At $T = 50$ mK, $B = 0$ with $V_{\mathrm{bg}} = -30$ V applied, its critical currents $I_c = I_{c+} = |I_{c-}| = 205\,\mathrm{nA}$ (Figure S5(d)), as there is no SDE at $B = 0$. We can compare this value to Devices A-C: (1) Device A, $I_c(B = 0) = 212\,\mathrm{nA}$; (2) Device B, $I_c(B = 0) = 198\,\mathrm{nA}$; (3) Device C, $I_c(B = 0) = 220\,\mathrm{nA}$, which are all measured at $T = 50$ mK with $V_{\mathrm{bg}} = -30$ V applied (main text Figure 2(d)(e)(f)). For Device D and E: (4) Device D, $I_c(B = 0) = 132\,\mathrm{nA}$ (Figure S13(b)); (5) Device E, $I_c(B = 0) = 120\,\mathrm{nA}$ (Figure S14(b)), both of which are measured at $T = 50$ mK with $V_{\mathrm{bg}} = -40$ V.

Under similar measurement conditions, the averaged I_c of WLs A-E is $\langle I_c(B = 0)\rangle_{A-E} =$ 175 nA, which is 85% of the I_c of Device F. If we nAïvely assume that the measured I_c is directly proportional to w_{WL}, then the averaged width of WLs A-E $\langle w_{\mathrm{WL}}\rangle_{A-E} \approx 85\% \times w_{\mathrm{WL,F}} \approx 51$ nm. We note the above argument may not hold in a 2D superconducting system with $w_{\mathrm{WL}} \approx 50$ nm$> \xi_{\mathrm{GL}} \approx 20$ nm (see Figure S6 for calibration of ξ_{GL}), where dissipation is governed by the entrance/nucleation of vortices. Nonetheless, I_c is still a monotonic function of w_{WL} in 2D. Since $\langle I_c\rangle_{A-E} = 85\%\ I_{c,F}$, we can still argue that $\langle w_{\mathrm{WL}}\rangle_{A-E}$ is a bit less than $w_{\mathrm{WL,F}} \approx 60\,\mathrm{nm}$. Thus, 50 nm is a credible expectation for the width of a typical WL created by the "cutting + bridging" lithographic process.

Moreover, by using $w_{\mathrm{WL}} = 50$ nm in the TDGL simulation, the calculated I_c arrives at $I_c(B = 0) = 190$ nA (main text Figure 4(g)), very close to the $\langle I_c(B = 0)\rangle_{A-E} = 175$ nA. In conclusion, we believe $l_{\mathrm{WL}} = 200$ nm and $w_{\mathrm{WL}} = 50$ nm to be a credible estimation for our WL dimensions.

Supplementary Note S3: Dependence of $I_c(B)$ and $\eta(B)$ on measurement configurations

Each of Devices A-C has 6 leads that can be used as either current leads or voltage leads during I-V measurements. Eight different measurement configurations have been used to probe Devices A-C, which are listed in Figure S7. $I_{c\pm}(B)$ of Device A changes subtly when measured by different configurations (Figure S8), with its η always lying within $\pm 4\%$. Meanwhile, $I_{c\pm}(B)$ patterns of Devices B and C shows obvious change upon switching configurations (Figure S9, Figure S10), as does the corresponding $\eta(B)$. Despite the remarkable changes in the specific $\eta(B)$ pattern, SDE in Device B does not switch sign, maintaining $\eta(B < 0) > 0$ and $\eta(B > 0) < 0$ under all configurations (Figure S9). The same argument holds for Device C, which always has $\eta(B < 0) < 0$ and $\eta(B > 0) > 0$ (Figure S10).

TDGL simulation provides qualitative explanations for the dependence of $I_c(B)$ and $\eta(B)$ on the choice of current leads. We simulate the device shown in main text Figure 4(a) again in Figure S11, the difference being that the current leads are relocated to the right edge in the configuration in Figure S11(a), and to the left edge in the configuration in Figure S11(b). Current density $\boldsymbol{K}$ is simulated under $B = -2000$ oe and $I = +150$ nA (Figure S11(c)(d)), same as the condition of main text Figure 4(b). Distribution of $\boldsymbol{K}$ changes upon switching to different current leads, so does the number and location of static vortices in the 2d channel (Figure S11(c) vs (d)). The right-sided current leads result in mobile vortex entry and dissipation (blue curves, Figure S11(e)). Meanwhile the left-sided current leads create a current profile that is less effective at forcing vortex entry near the WL (a higher surface barrier), causing absence of dissipation (orange curves, Figure S11(e)).

Simulated $I - V$ curves at $B = -2000\,\mathrm{Oe}$ give $I_{c+} = 156\,\mathrm{nA}$ for right-sided current leads, a close $I_{c+} = 157.5\,\mathrm{nA}$ for the top/bottom current leads in Figure 4(a), and an increased $I_{c+} = 163.5\,\mathrm{nA}$ for left-sided leads (Figure S11(f)). Under negative bias, these three configurations have the same $I_{c-} = -172.5\,\mathrm{nA}$ (Figure S11(g)). Simulated diode

efficiency $\eta = +5.0\%\,, +4.5\%\,, +2.7\%$ for the right-sided, top/bottom and left-sided current leads, which differ in magnitude but maintain the same sign, consistent with the experimental observation in Figure S9 and Figure S10. In conclusion, according to the TDGL simulation, choosing different current leads can change I_c due to the alteration of current density and vortex surface barriers. We also note the thermalization time $T_{therm} = 80\tau_0$ used to simulate $I - V$ curves results in a slightly higher I_c in Figure S11(f) as compared to Figure S11(e), where only one current value is simulated with prolonged thermalization step $T_{therm} = 550\tau_0$ for the device to fully stabilize (see Methods Section).

Another possible but less straightforward reason for the dependence of $I_c(B)$ on the lead configuration is the phase slips occuring within the current leads, which may induce premature phase slips in the WL through certain nonlocal interactions (such as heating or AC Josephson effect).

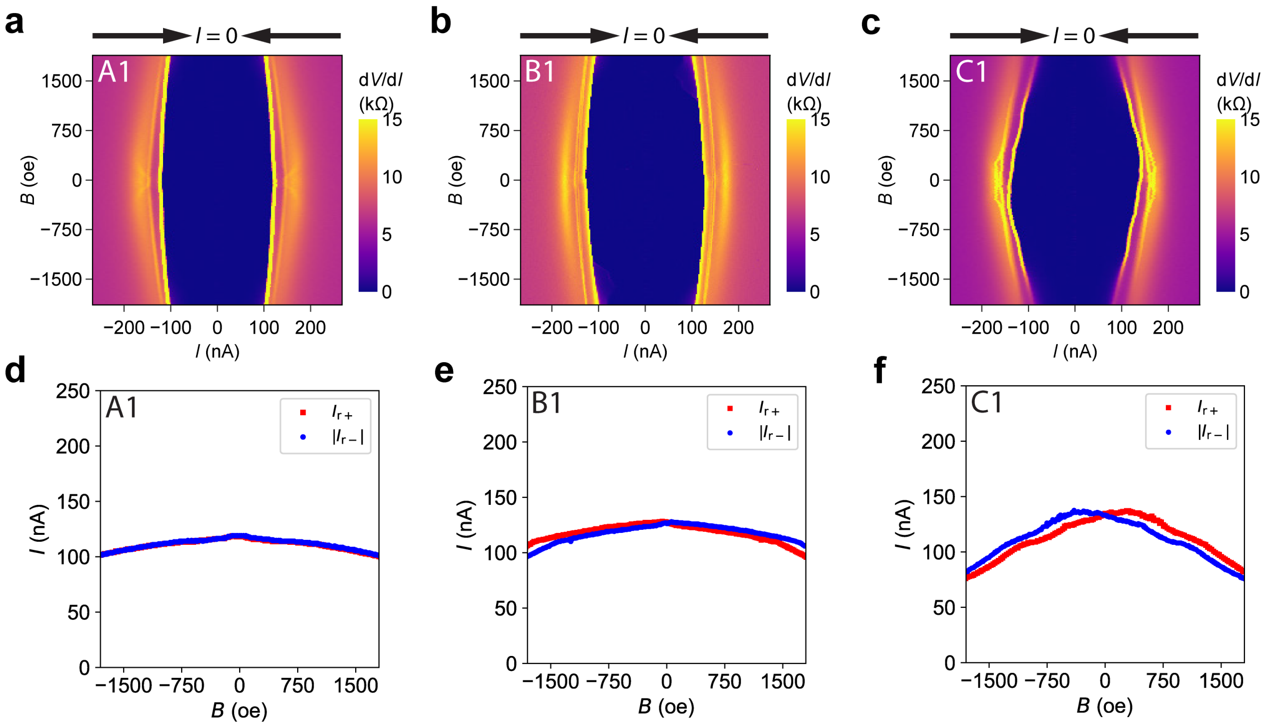

Figure S1: Retrapping currents of Devices A-C vs magnetic field. **(a)(b)(c)** Intensity plots of differential resistance dV/dI vs I vs B of Devices A, B and C. In these plots, current I sweeps from $|I| > 0$ to $I = 0$, as indicated by the black arrows above each plot. **(d)(e)(f)** Extracted retrapping currents $I_{r\pm}$ of Devices A-C. These plots are from the same dataset as main text Figure 2, which was taken at $T = 50$ mK with a backgate voltage $V_{\rm bg} = -30$ V applied on Devices A-C.

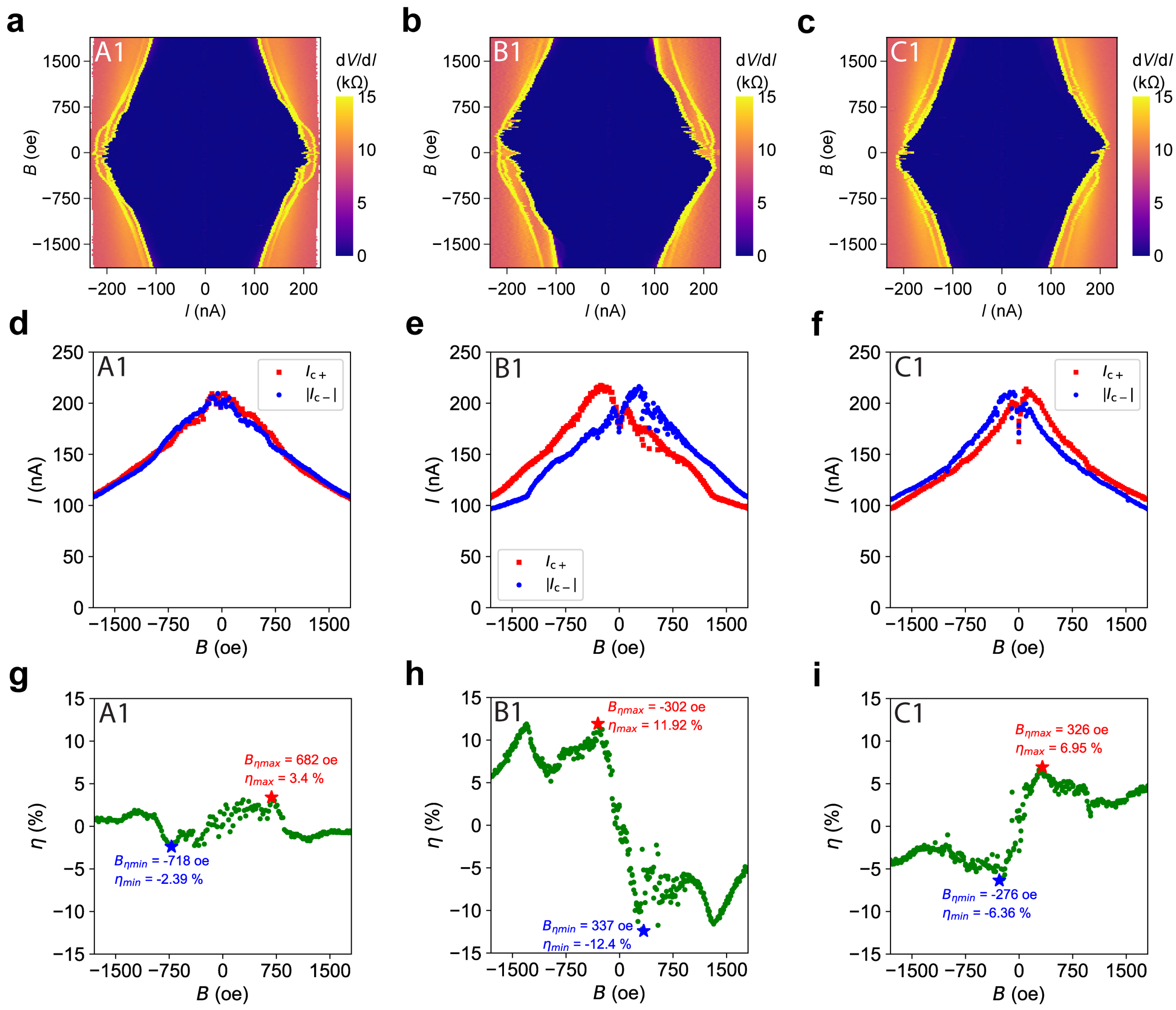

Figure S2: Magnetic field sweep of Devices A-C taken at $V_{\rm bg} = -55$ V. **(a)(b)(c)** Intensity plots of dV/dI vs I vs B of Devices A, B and C. **(d)(e)(f)** Switching current $I_{c\pm}$ of Devices A-C as a function of B. **(g)(h)(i)** Diode efficiency η of Devices A-C as a function of B, with the locations of $\eta_{\rm max(min)}$ labeled. All plots in this figure were taken at $T = 50$ mK with $V_{\rm bg} = -55$ V applied on Devices A-C.

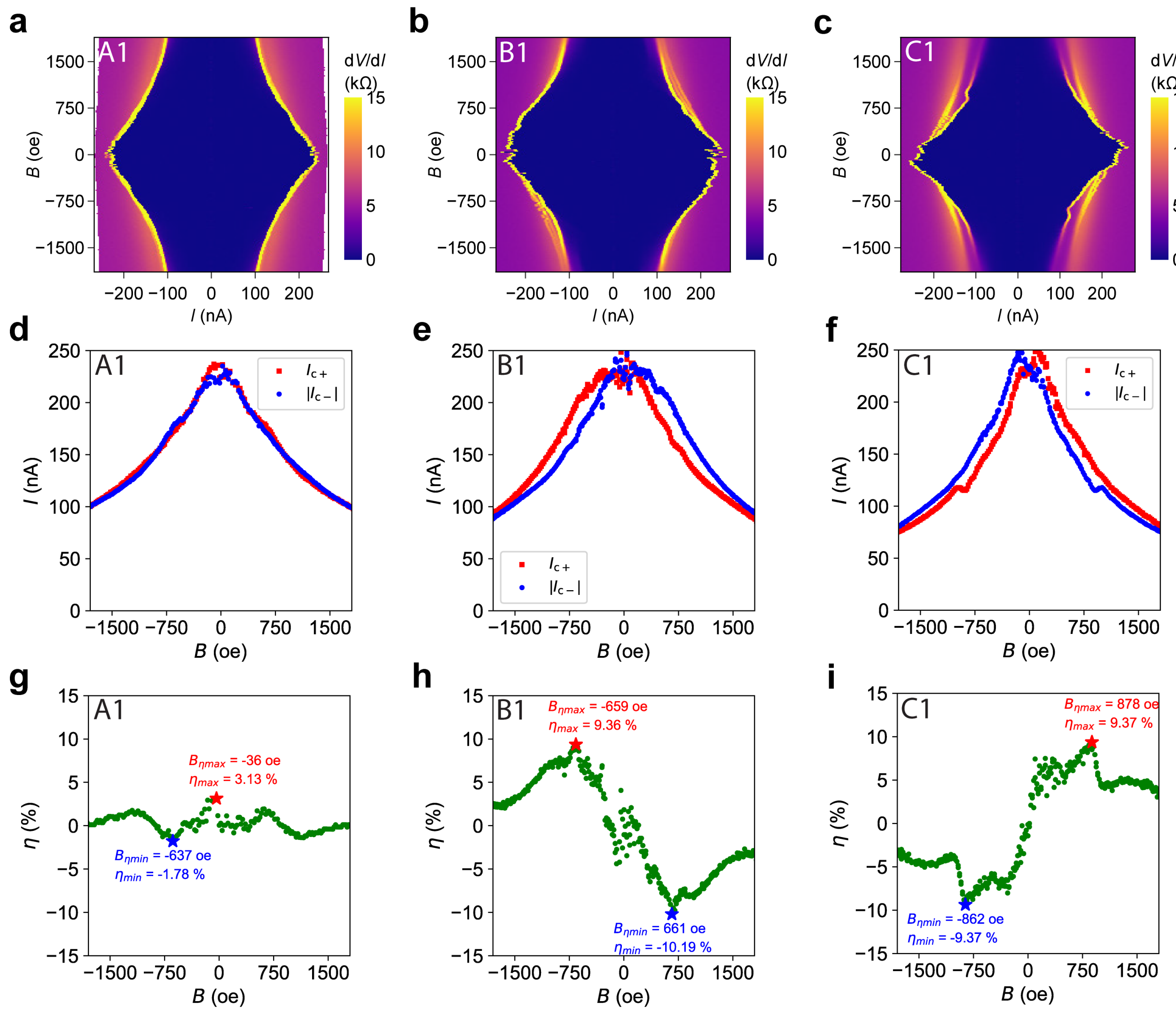

Figure S3: Magnetic field sweep of Devices A-C taken at $V_{\text{bg}} = 0$ V. **(a)(b)(c)** Intensity plots of dV/dI vs I vs B of Devices A, B and C. **(d)(e)(f)** Switching current $I_{c\pm}$ of Devices A-C as a function of B. **(g)(h)(i)** Diode efficiency η of Devices A-C as a function of B, with the locations of $\eta_{\max(\min)}$ labeled. All plots in this figure were taken at $T = 50$ mK with backgate grounded ($V_{\text{bg}} = 0$ V).

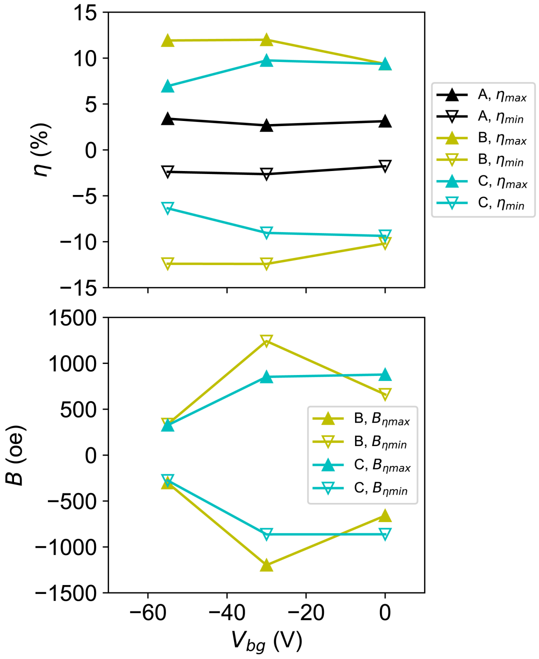

Figure S4: Backgate dependence of diode efficiency of Devices A-C. Optimal diode efficiency $\eta_{\max(\min)}$ (top) and corresponding optimal magnetic field $B_{\eta\min(\eta\max)}$ (bottom) are plotted as a function of V_{bg} applied on the sample. The datapoints are extracted from main text Figure 2(g)-(i) as well as Figure S2(g)-(i) and Figure S3(g)-(i).

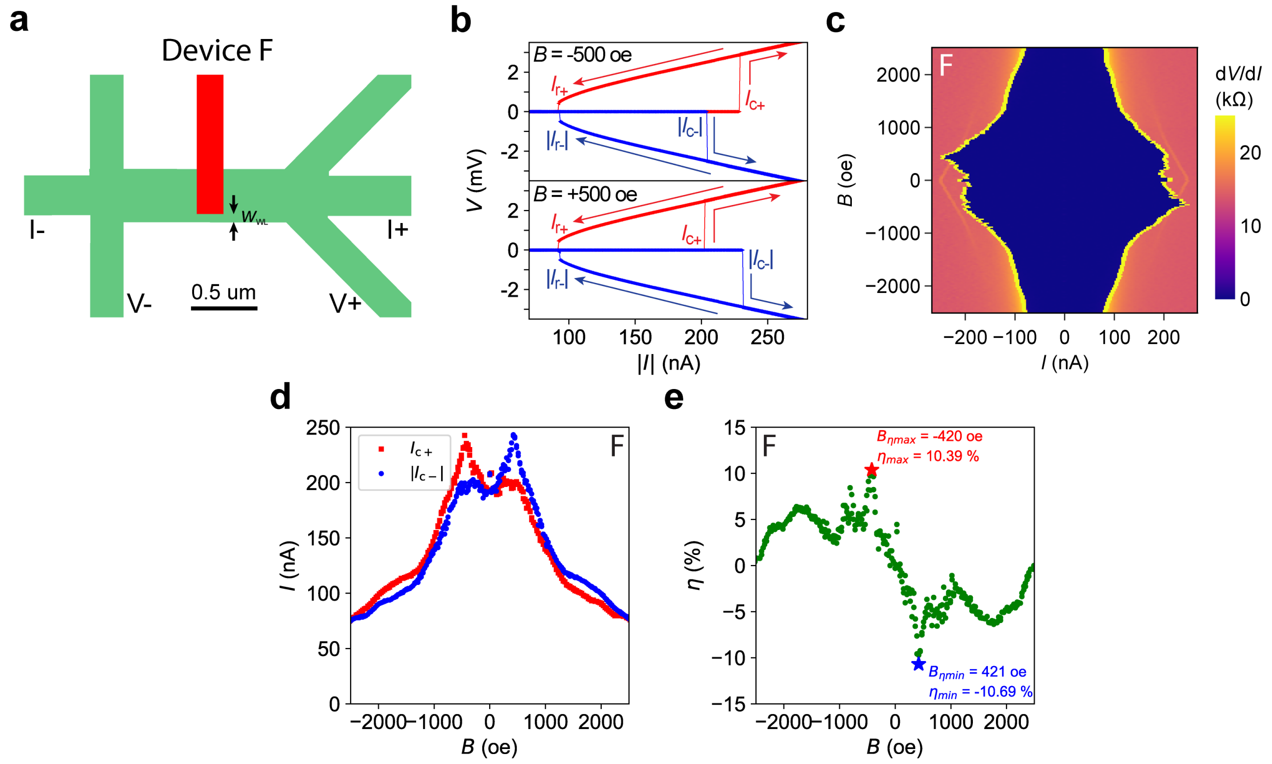

Figure S5: Supercurrent Diode Device F. **(a)** Layout of Device F. Instead of cutting the 1D channel completely into the left and right halves and then writing the WL like Devices A-E, the 2D channel is only partially cut to leave a 60 nm-wide conducting path near the bottom edge. In this way we effectively create a WL with $w_{\mathrm{WL}} \approx 60$ nm. **(b)** I-V measurements of Device F at $B = \pm 500$ Oe, where arrows indicate the I sweep direction. **(c)(d)(e)** dV/dI vs I vs B intensity plot, $I_{c\pm}$ vs B and η vs B plots of Device F. All plots in this figure were taken at $T = 50$ mK, with $V_{\mathrm{bg}} = -30$ V applied on the sample.

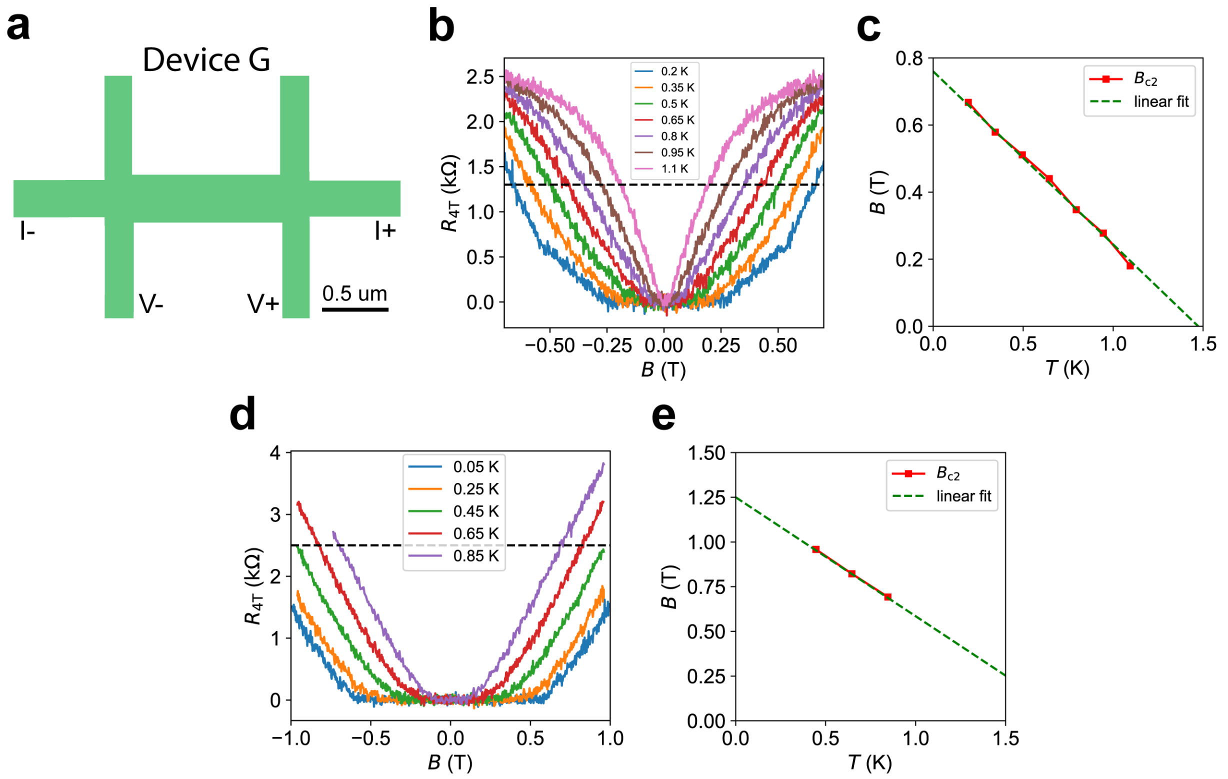

Figure S6: Reference Hallbar Device G. **(a)** Layout of Hallbar. **(b)** Four-terminal resistance $R_{4\mathrm{T}}$ as a function of B measured at different temperatures from 0.2 K to 1.1 K, with backgate grounded ($V_{\mathrm{bg}} = 0$ V). The black dashed line indicates half of the normal state resistance $R_N/2$. **(c)** Upper critical field B_{c2} as a function of T at $V_{\mathrm{bg}} = 0$ V. B_{c2} at each temperature is extracted at $R_{4\mathrm{T}} = R_N/2$ from panel (b). Performing linear fit using $B_{c2}(T) = \frac{\Phi_0}{2\pi\xi_{\mathrm{GL}}^2}(1 - T/T_c)$ gives $B_{c2}(0) = 0.73$ T and $\xi_{\mathrm{GL}} = 21.2$ nm. **(d)** $R_{4\mathrm{T}}$ as a function of B measured at different temperatures from 0.05 K to 0.85 K, while applying $V_{\mathrm{bg}} = -60$ V on the sample. **(e)** Upper critical field B_{c2} as a function of T at $V_{\mathrm{bg}} = -60$ V, extracted from panel (d). Linear fit gives $B_{c2}(0) = 1.25$ T and $\xi_{\mathrm{GL}} = 16.2$ nm.

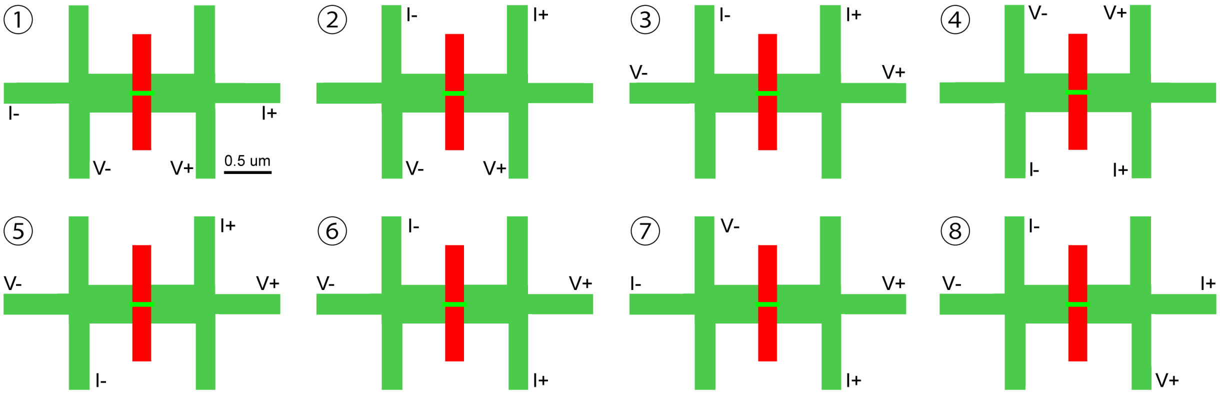

Figure S7: Measurement configurations of Devices A-C. Each configuration is labeled with a number at its top right corner, which is referred to by I-V measurements of Devices A-C. In each configuration, current source/drain are labeled by $I+/I-$, while the two voltage leads are labeled by $V+/V-$. We note that positive current $I > 0$ always flow through the WL from right to left in all configurations.

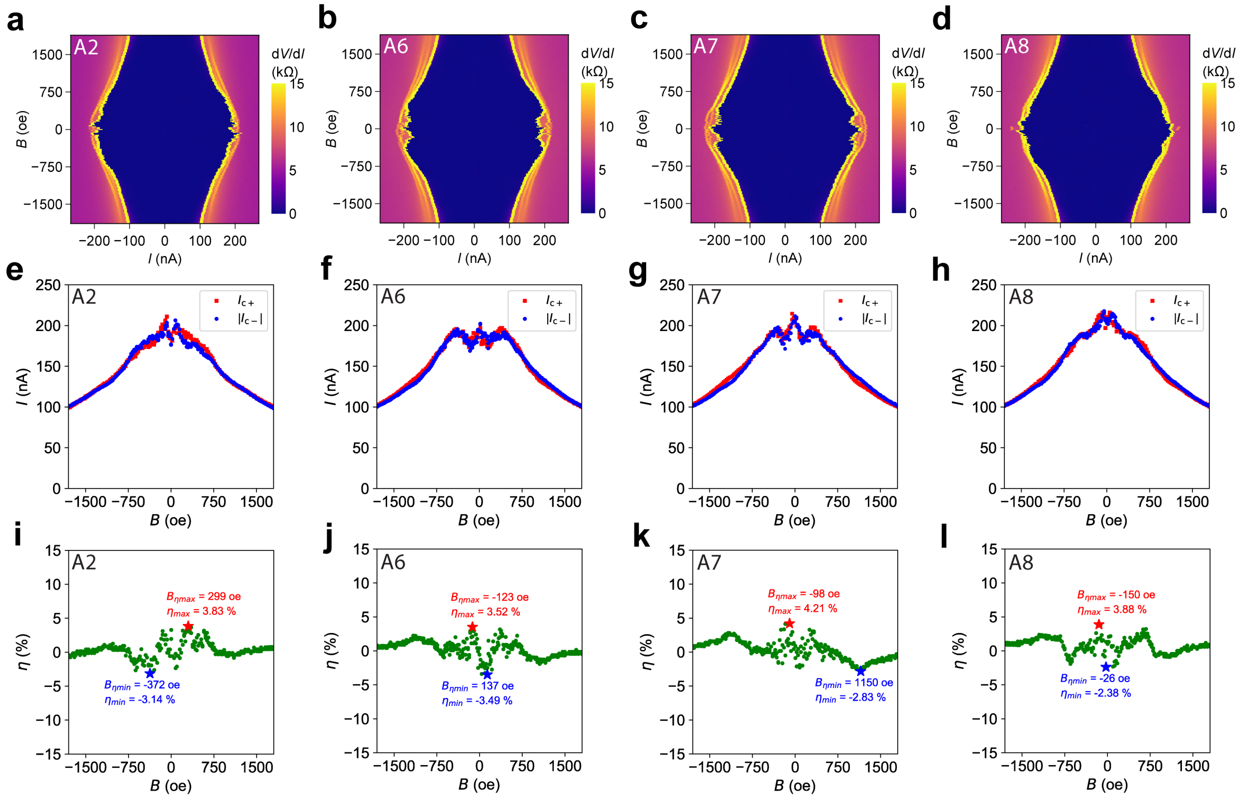

Figure S8: Device A under different measurement configurations. **(a)-(d)** dV/dI vs I vs B plots of Device A, with corresponding measurement configuration labeled at the top left corner of each plot (refer to Figure S7). **(e)-(h)** I_c vs B of Device A measured at different configurations. **(i)-(l)** Extracted η vs B of Device A at different configurations. All plots in this figure were taken at $T = 50$ mK with $V_{\rm bg} = -30$ V applied on Device A.

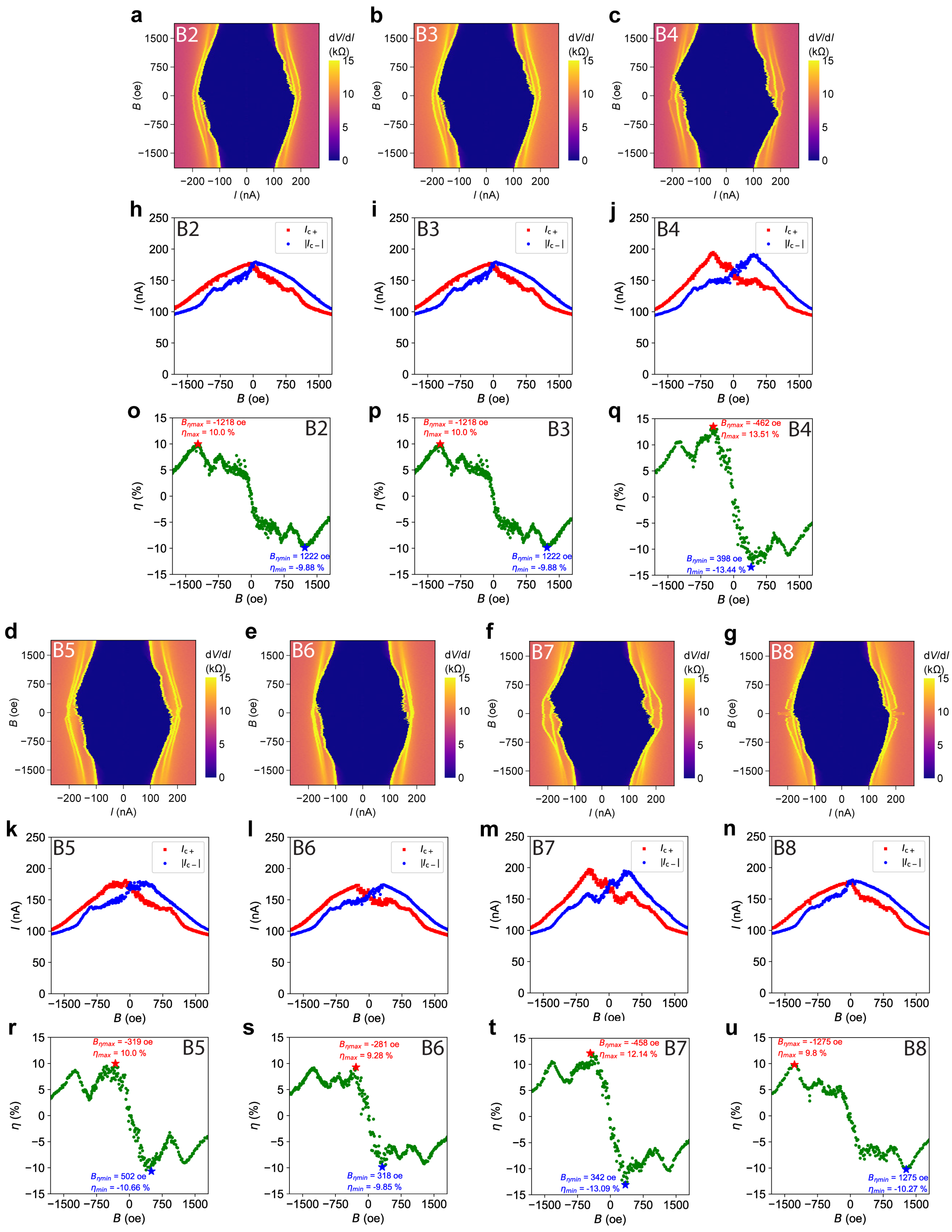

Figure S9: Device B under different measurement configurations. **(a)-(g)** dV/dI vs I vs B plots of Device B, with corresponding measurement configuration labeled at the top left corner of each plot (refer to Figure S7). **(h)-(n)** I_c vs B of Device B measured at different configurations. **(o)-(u)** Extracted η vs B of Device B at different configurations. All plots in this figure were taken at $T = 50$ mK with $V_{\rm bg} = -55$ V applied on Device B.

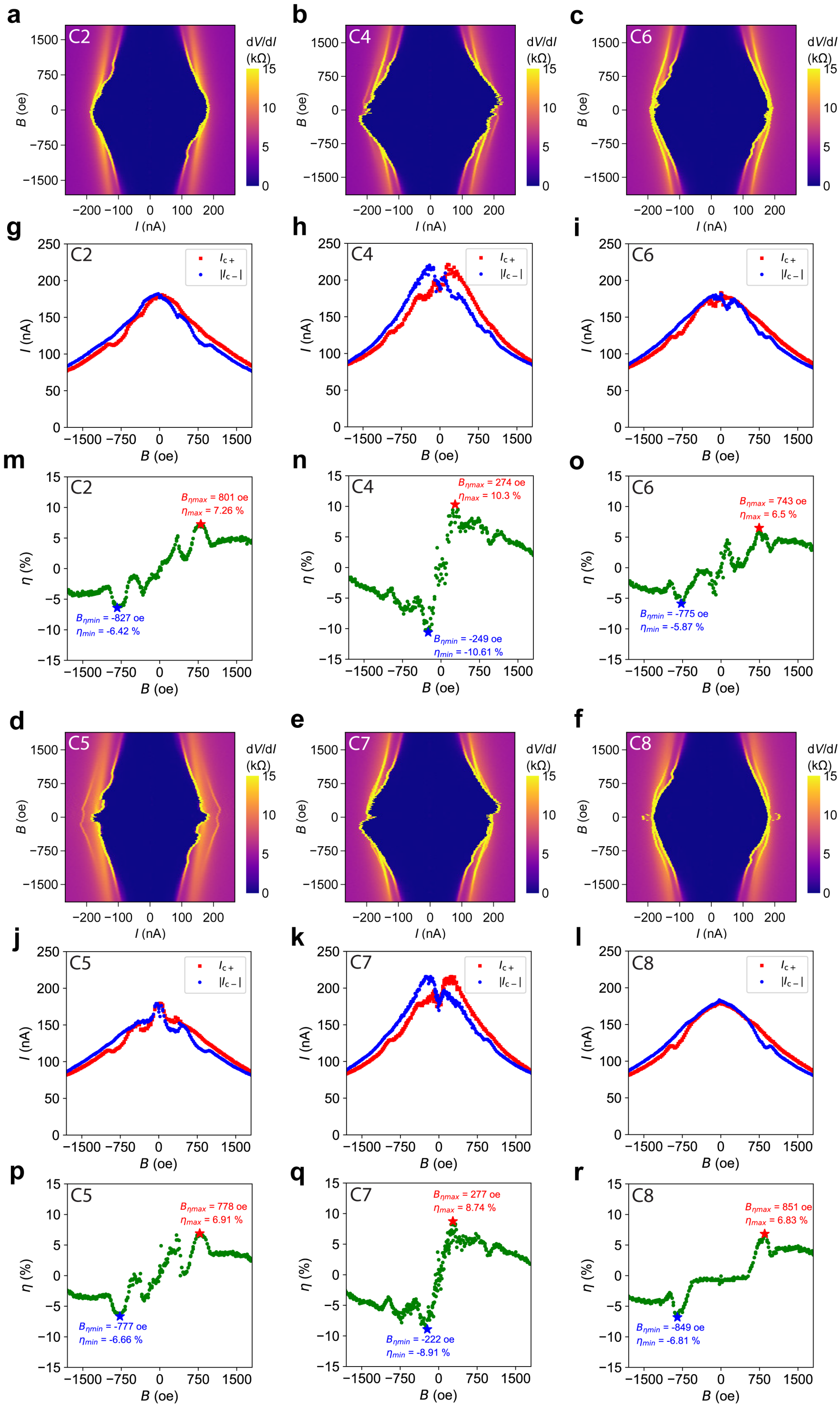

Figure S10: Device C under different measurement configurations. **(a)-(f)** dV/dI vs I vs B plots of Device C, with corresponding measurement configuration labeled at the top left corner of each plot (refer to Figure S7). **(g)-(l)** I_c vs B of Device C measured at different configurations. **(m)-(r)** Extracted η vs B of Device C at different configurations. All plots in this figure were taken at $T = 50$ mK with $V_{\mathrm{bg}} = -30$ V applied on Device C.

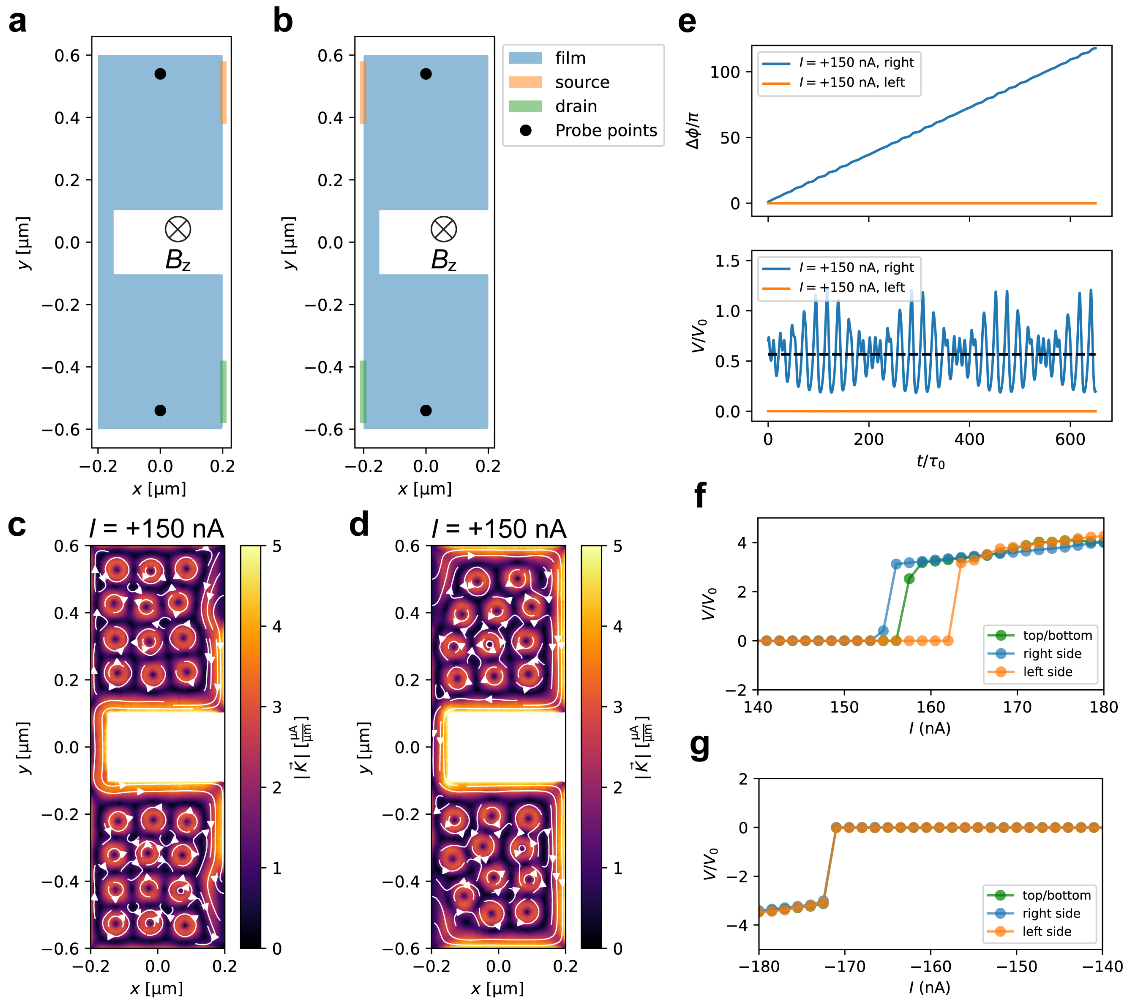

Figure S11: TDGL simulation of the WL with different current sources and drains. **(a)** The simulated device is exactly the same as main text Figure 4(a), except here the current source and drain are put on the right edge of the device. **(b)** In this configuration the current source and drain are put on the left side. **(c)(d)** Current density $\boldsymbol{K}$ simulated under $B = -2000\,\mathrm{Oe}$ field and $I = +150$ nA bias, using the configuration in panel (a) and (b) respectively. **(e)** Evolution of phase difference $\Delta\phi(t)$ and voltage $V(t)$. Black dashed line: time-averaged voltage of the configuration in (a). **(f)(g)** Simulated $I-V$ curves at $B = -2000\,\mathrm{Oe}$ using different configurations. Green curve is simulated using the configuration in main text Figure 4(a) where current source and drain are on the top and bottom edges. Blue and orange curves are simulated using the configurations in panel (a) (right-sided source and drain) and (b) (left-sided source and drain), respectively.

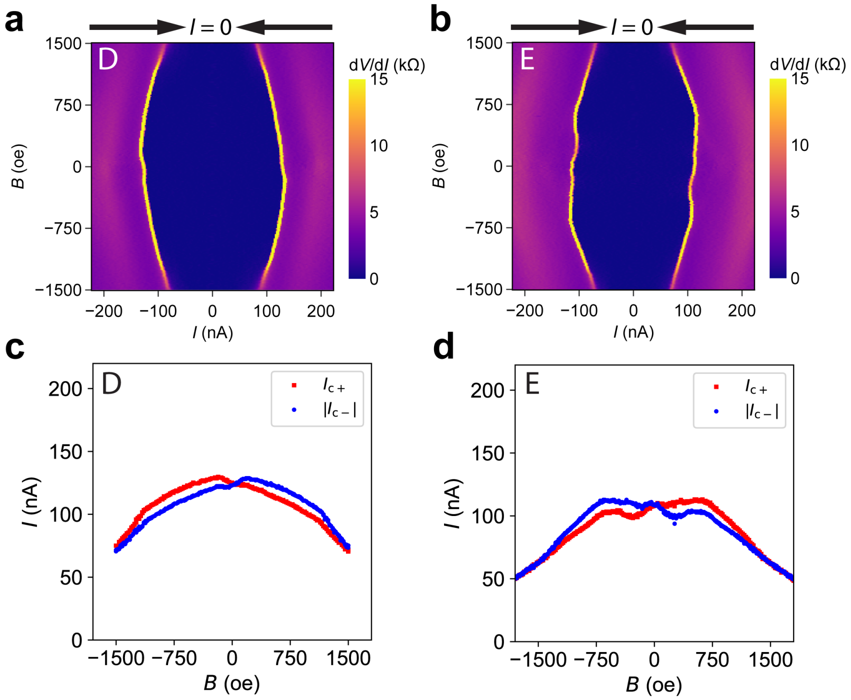

Figure S12: Retrapping currents of Devices D and E vs magnetic field. **(a)(b)** Intensity plots of differential resistance dV/dI vs I vs B of Devices D and E. In these plots, current I sweeps from $|I| > 0$ to $I = 0$, as indicated by the black arrows above each plot. **(c)(d)** Extracted retrapping currents $I_{r\pm}$ of Devices D and E. These plots are from the same dataset as main text Figure 3, which was taken at $T = 50$ mK with backgate grounded ($V_{\text{bg}} = 0$ V).

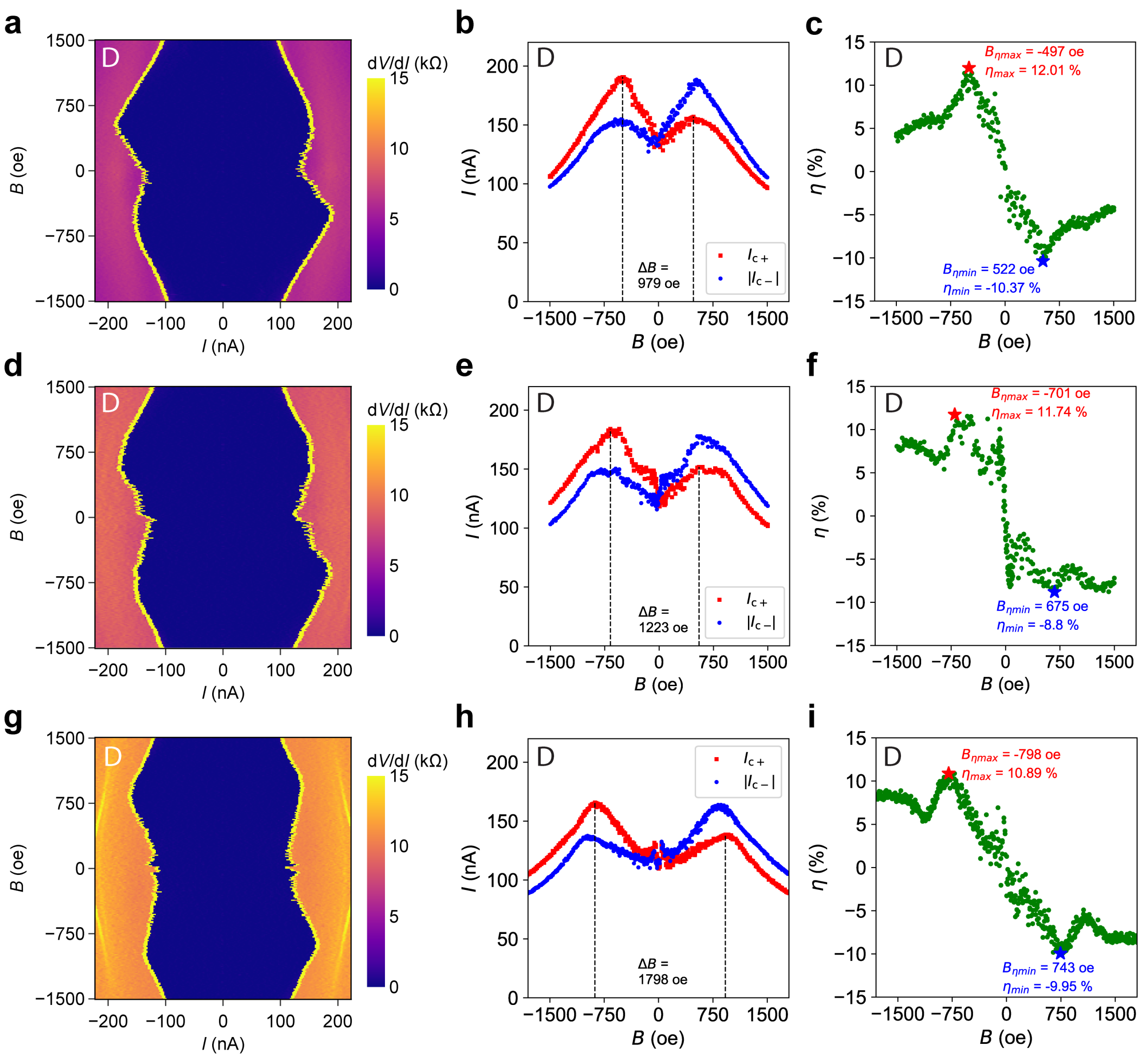

Figure S13: Device D measured at different backgate voltages. **(a)(b)(c)** dV/dI vs I vs B intensity plot, $I_{c\pm}$ vs B and η vs B relations of Device D, measured at $V_{\text{bg}} = -40$ V. The splitting ΔB between the two I_c maxima is labeled, as well as the location of $\eta_{\max}$ and $\eta_{\min}$. **(d)(e)(f)** dV/dI vs I vs B intensity plot, $I_{c\pm}$ vs B and η vs B relations of Device D, measured at $V_{\text{bg}} = -60$ V. **(g)(h)(i)** dV/dI vs I vs B intensity plot, $I_{c\pm}$ vs B and η vs B relations of Device D, measured at $V_{\text{bg}} = -80$ V. All plots in this figure were taken at $T = 50$ mK.

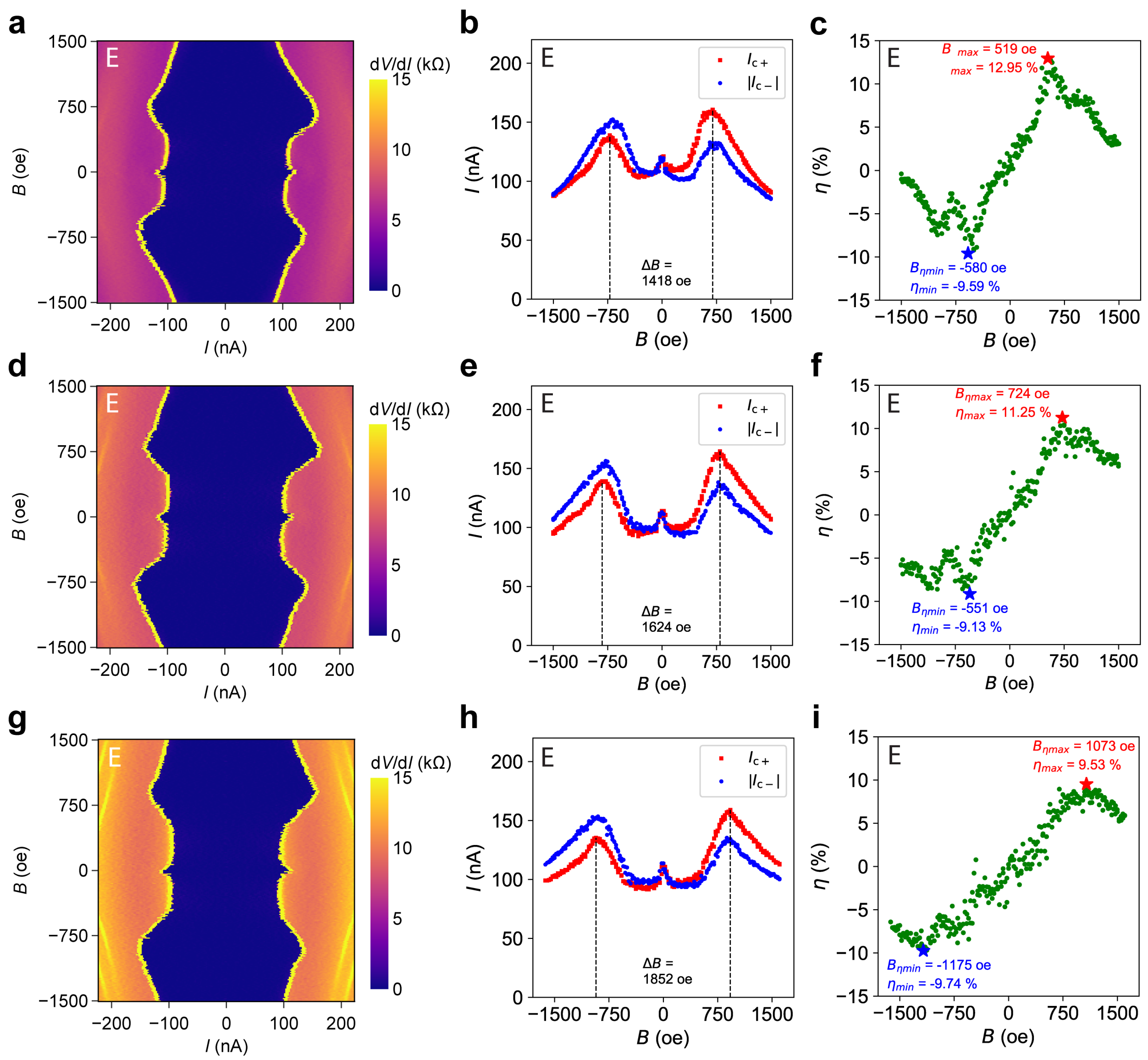

Figure S14: Device E measured at different backgate voltages. **(a)(b)(c)** dV/dI vs I vs B intensity plot, $I_{c\pm}$ vs B and η vs B relations of Device E, measured at $V_{\rm bg} = -40$ V. The splitting ΔB between the two I_c maxima is labeled, as well as the location of $\eta_{\rm max}$ and $\eta_{\rm min}$. **(d)(e)(f)** dV/dI vs I vs B intensity plot, $I_{c\pm}$ vs B and η vs B relations of Device E, measured at $V_{\rm bg} = -60$ V. **(g)(h)(i)** dV/dI vs I vs B intensity plot, $I_{c\pm}$ vs B and η vs B relations of Device E, measured at $V_{\rm bg} = -80$ V. All plots in this figure were taken at $T = 50$ mK.

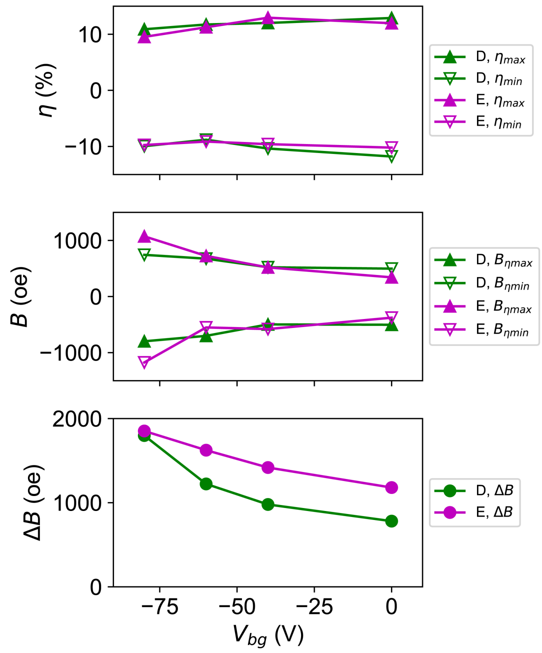

Figure S15: Backgate dependence of diode efficiency of Devices D and E. Optimal diode efficiency $\eta_{\max(\min)}$ (top) and corresponding optimal magnetic field $B_{\eta\min(\eta\max)}$ (middle) are plotted as a function of V_{bg} applied on the sample. The datapoints are extracted from main text Figure 3(f)(i) as well as Figure S13(c)(f)(i) and Figure S14(c)(f)(i). At $T = 50$ mK, I_c vs B relation of Device D or E resembles a M-shape, with ΔB splitting the two I_c peaks. Here ΔB of Device D and E are plotted as a function of V_{bg} in the bottom panel.

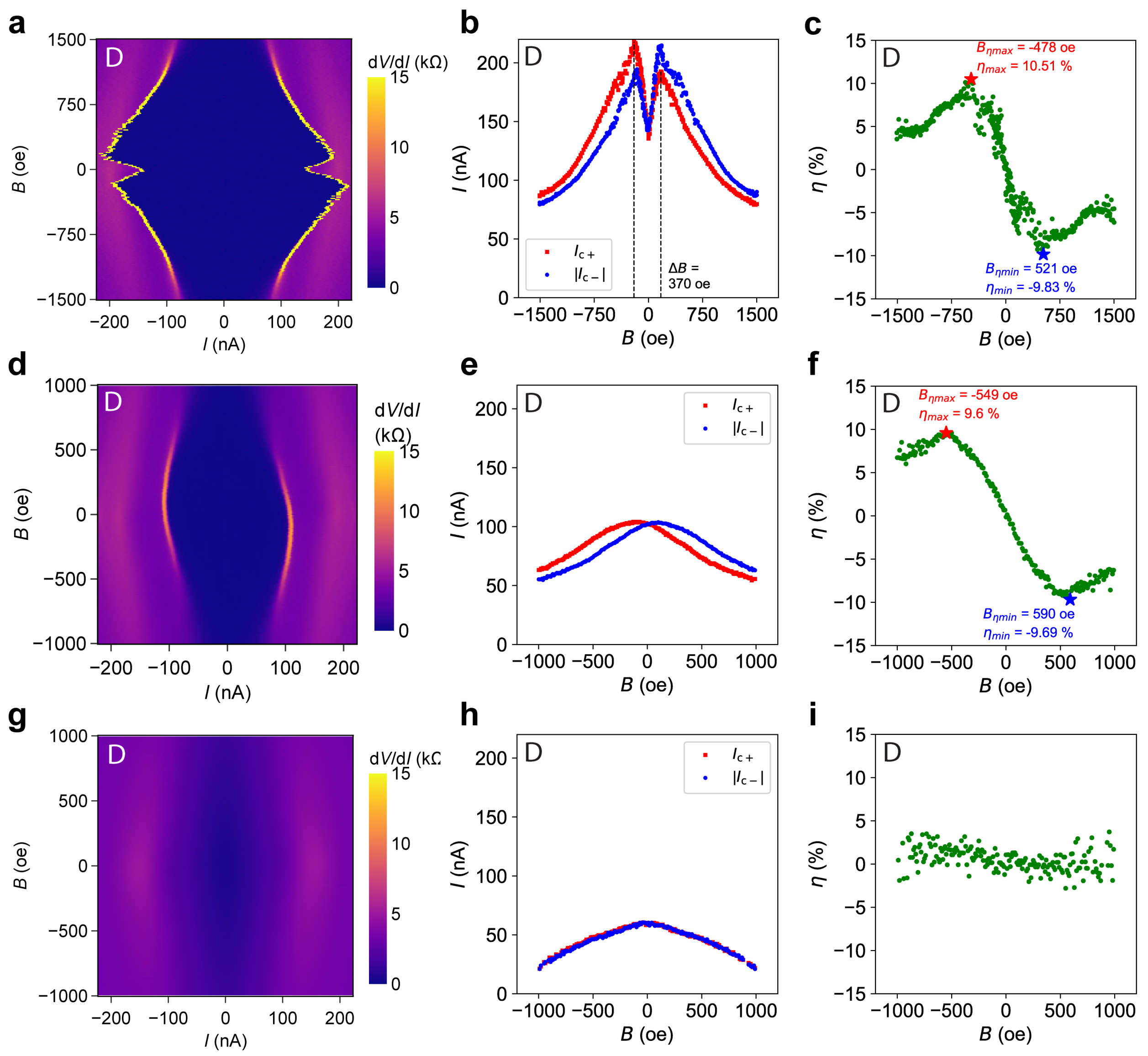

Figure S16: Temperature dependence of Device D. **(a)(b)(c)** dV/dI vs I vs B intensity plot, $I_{c\pm}$ vs B and η vs B relations of Device D, measured at $T = 500$ mK. The splitting ΔB between the two I_c maxima is labeled, as well as the location of $\eta_{\max}$ and $\eta_{\min}$. **(d)(e)(f)** dV/dI vs I vs B intensity plot, $I_{c\pm}$ vs B and η vs B relations of Device D, measured at $T = 900$ mK. M-shaped I_c vs B feature is suppressed at this temperature. **(g)(h)(i)** dV/dI vs I vs B intensity plot, $I_{c\pm}$ vs B and η vs B relations of Device D, measured at $T = 1.2$ K. All plots in this figure were taken with backgate grounded ($V_{\text{bg}} = 0$ V).
